# Effect of Ionomer–Solvent Interactions in PFSA Dispersions: Dispersion Viscosity

**DOI:** 10.1021/acs.macromol.5c00637

**Published:** 2025-12-13

**Authors:** Melissa Novy, Denis Duchesne, Gregg Dahlke, Lisa P. Chen, Robert B. Moore

**Affiliations:** † Department of Chemistry, Macromolecules Innovation Institute, Virginia Tech, Blacksburg, Virginia 24061, United States; ‡ 3M Advanced Materials Division, 3M Center, Building 280-1W-03, St. Paul, Minnesota 55144, United States

## Abstract

Perfluorosulfonic acid ionomer (PFSA) dispersions are essential to the coating processes used to fabricate membranes, catalyst layers, and thin films for hydrogen fuel cell and water electrolyzer applications. The PFSA dispersion viscosity can significantly affect coating parameters including wetting, leveling, and compatibility with coating equipment. The effect of PFSA concentration, chemical structure, and solvent composition on dispersion viscosity is examined as a function of five different PFSAs and three different binary alcohol–water solvent systems, using n-propanol, isopropanol, or ethanol as the alcohol. The zero-shear viscosity, η_0_, is observed to increase with decreasing side chain length, increasing side chain content, and increasing alcohol concentration in the binary alcohol–water solvent. A direct comparison is made between the PFSA colloidal morphology discussed in a previous publication by the present authors and η_0_. At a fixed, nondilute PFSA concentration, two regimes of weak and strong dependence of η_0_ on alcohol concentration in the solvent are identified. In the regime where η_0_ weakly depends on alcohol concentration, an increase in η_0_ is associated with an increase in the aggregate surface area normalized by side chain content. The orders of magnitude increases in η_0_ with increasing alcohol concentration in the regime of strong dependence of η_0_ are attributed to both aggregate morphology and interaggregate ionic associations. By independently considering the PFSA side chain and backbone solubility parameters, two regimes corresponding to relatively favorable solvent-side chain and relatively favorable solvent-backbone interactions are defined as a function of alcohol–water solvent composition. An analysis of PFSA-solvent interaction parameters shows that interaggregate ionic associations occur when solvent-side chain interactions are unfavorable relative to solvent-backbone interactions—for example, at high alcohol concentrations in the solvent. The alcohol concentration corresponding to the crossover between the weak and strong regimes of η_0_ is found to agree within ±5 wt % alcohol with the crossover between the regimes of favorable solvent-side chain and favorable solvent-backbone interactions.

## Graphical abstract

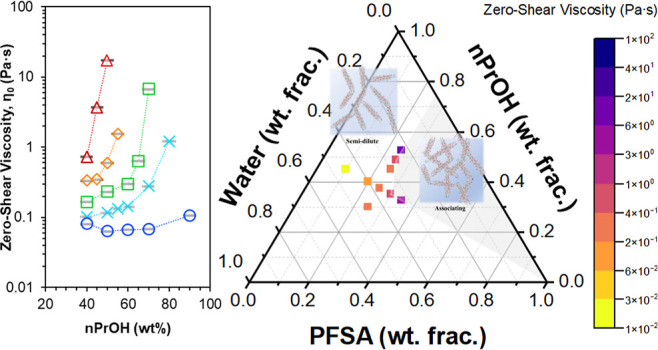

## Introduction

Perfluorosulfonic acid ionomers (PFSAs) are the current benchmark materials used in the solid electrolyte membrane and catalyst layers of proton-exchange membrane (PEM) fuel cells and water electrolyzers. PFSAs are copolymers of tetrafluoroethylene (TFE) and perfluorosulfonic acid vinyl ether, where the TFE units comprise the backbone and the perfluorosulfonic acid vinyl ether groups act as side chains terminating in sulfonic acid groups. The PFSA copolymer composition is commonly represented by equivalent weight (EW) in units of grams of polymer per moles of sulfonic acid groups. The general chemical structures of three commercially available PFSAs are shown in Figure 1a, where the designation C2, C4, or LSC refers to the length of the side chain and n represents the average number of TFE repeat units between side chains. In protic solvents, PFSAs are known to form dispersions of rodlike aggregates of chains, where the hydrophilic sulfonic acid side chains are predominantly located at the solvent-aggregate interface and the hydrophobic backbone forms the aggregate core.^1–8^ Many studies have found that improving the compatibility between the hydrophobic backbone and solvent, such as in binary alcohol–water systems at high alcohol concentrations, results in decreased aggregation.^8–13^

**Figure 1:**
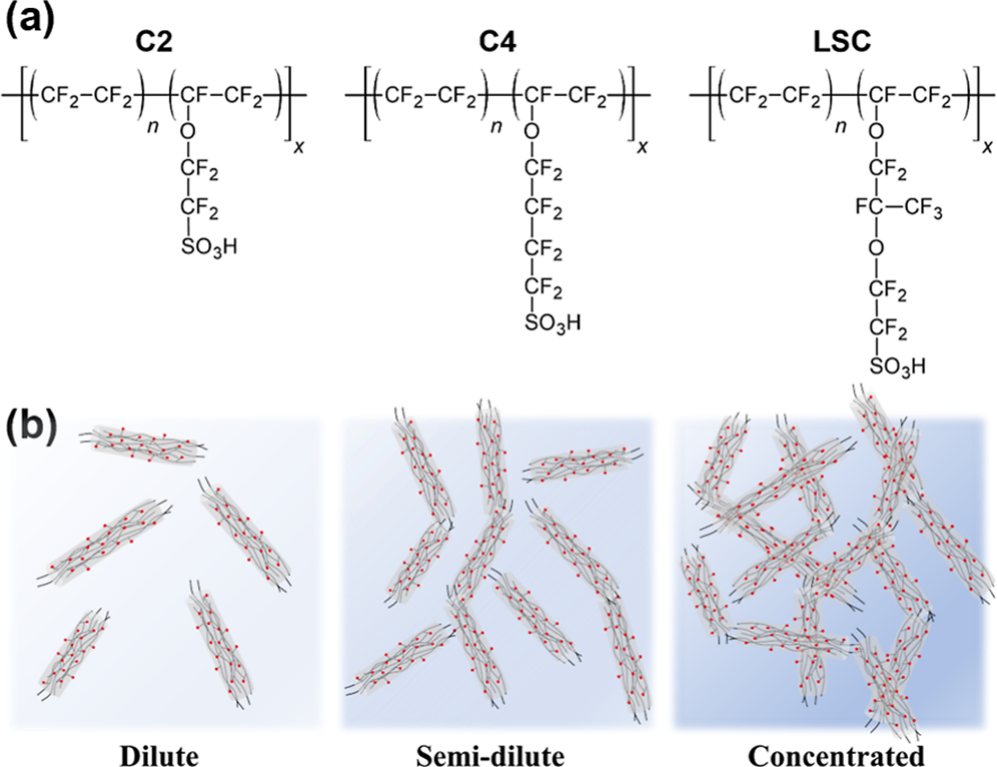
(a) General chemical structures of three commercially available PFSAs. (b) Schematic of the rodlike aggregate morphology of PFSAs in protic solvents.

Aggregation in PFSA dispersions is also strongly dependent on ionomer concentration. In the dilute regime in protic solvents, hydrophobic aggregation of PFSA chains results in isolated primary aggregates.^4,14–16^ In the semidilute regime, secondary aggregation, i.e., aggregates of aggregates, has been observed through small-angle scattering, electron spin resonance, NMR, cryo-microscopy, and modeling.^7,9,13,17,18^ However, there is no literature consensus on whether ionic interaggregate associations and/or interaggregate chain entanglements result in secondary aggregation in the semidilute regime. For example, Kim et al. and Szajdzinska-Pietek and Schlick proposed that secondary aggregation in protic solvents occurs due to physical entanglements of chains between aggregates.^7,17^ Ngo et al. suggested that interaggregate ionic associations occur in low alcohol content (balance water) solvents.^9^ In contrast, Khandavalli et al. attributed interaggregate ionic associations in high alcohol content (balance water) solvents to the condensation of sulfonate groups as the solvent dielectric constant decreased with decreasing water content.^12^ Work on coalescence of PFSA dispersions into films has shown that the decreasing distance between aggregates as PFSA concentration increases due to solvent evaporation eventually results in the formation of electrostatically cross-linked, sulfonate-rich domains.^1,17,19–23^ Thus, interaggregate ionic associations are expected to occur at sufficiently high PFSA concentrations, i.e., in the concentrated regime.^7,9,12,17^ A representation of the rodlike colloidal morphology of PFSA dispersions in protic solvents in different concentration regimes is shown in Figure 1b.

Industrial manufacture of PFSA membranes and catalyst layers commonly involves a roll-to-roll process where a PFSA dispersion or catalyst ink is coated onto a backing film.^24^The dispersion rheology is an important parameter of the coating techniques—including slot-die printing, screen printing, blade coating, and spin-coating^25^—used to fabricate PFSA membranes, thin films, and catalyst layers.^26^ Wet film thickness, leveling of the coating,^27–30^ and compatibility with coating equipment^31^ are all directly influenced by the rheological behavior of the dispersion. As the viscosity of polymer solutions is generally understood to be related to friction between the solvent and polymer chains or aggregates,^32,33^ it is reasonable to imagine that the PFSA concentration-dependent and solvent-dependent dispersion morphology affects the viscosity of the dispersion. PFSA dispersions in alcohol–water solvent from 0.1 to >20 wt % PFSA were found to obey viscosity-polymer concentration scaling laws derived from the concentration-dependent conformations of charged polymer chains in solution.^12,34^ These studies contributed to the identification of dilute, semidilute, and concentrated regimes of PFSA dispersions characterized by the distinct aggregation behavior described above. For example, studies of the polyelectrolyte effect in dilute PFSA dispersions in binary alcohol–water solvent attributed the increase in reduced viscosity with decreasing ionomer concentration to extension of the chains comprising the PFSA aggregates.^12,16,35^ Furthermore, shear-dependent dispersion viscosity measurements have been used to indirectly measure aggregation of catalyst particles in PFSA catalyst inks.^36–38^ Small-angle X-ray scattering (SAXS) and viscosity measurements of solutions of sulfonated polystyrene, a model ionomer, in mixtures of tetrahydrofuran and D_2_O found that higher solution viscosities were associated with chain extension observed by SAXS.^39^ These studies suggest that PFSA dispersion morphology and viscosity are connected. However, no studies to date have systematically investigated the influence of colloidal dispersion morphology on viscosity or mapped the viscosity behavior of PFSA dispersions at concentrations conventionally used to cast free-standing membranes, i.e., nondilute PFSA dispersions. In addition, much of the current literature on PFSA dispersions pertains only to Nafion, a prototypical long side chain (LSC) ionomer. Other commercially available but less studied ionomers include Aquivion, a brand of short side chain PFSAs (designated by C2), and the 3 M PFSA, a brand of medium side chain PFSA (designated by C4) (Figure 1a). In practical applications, the equivalent weight and/or side chain chemical structure of the PFSA is chosen for the design specifications of the PEM fuel cell or water electrolyzer.^40^

As industrial PFSA dispersion coating methods rely on equipment that operates within a specified optimal viscosity range,^31^ understanding the viscosity behavior of PFSA dispersions as a function of ionomer chemical structure and solvent composition can contribute to improving these processing methods. Furthermore, a detailed investigation of the relationship between solvent composition and colloidal morphology of PFSA dispersions may help to uncover connections to the morphology and properties of the resulting PFSA membranes and thin films. The colloidal morphology of nondilute PFSA dispersions was investigated by SAXS in a previous publication by the present authors.^8^ This morphology will be considered for direct comparison to viscosity measurements of the same dispersions in the present work. It is hypothesized that significant variations in PFSA dispersion zero-shear viscosities as a function of chemical structure and solvent composition are the result of changes in aggregate dimensions and interaggregate ionic associations. Additionally, the fundamental connections between PFSA chemical structure, solvent composition, aggregate morphology, and interaggregate associations are examined by an analysis of PFSA-solvent interaction parameters. This study appears to be the first to examine the behavior of both solvent-backbone and solvent-side chain interactions as a function of PFSA chemical structure and solvent composition. The results are found to be in good agreement with the colloidal PFSA morphologies observed by SAXS and the corresponding viscosities of the dispersions. Moreover, the interaction parameter analysis is demonstrated to be an indicator of different regimes of PFSA-solvent interactions, which result in distinct viscosity behaviors observed in both dilute and nondilute PFSA dispersions.

## Experimental Section

### Materials

Aquivion D83–24B (24 wt % 830 EW C2) aqueous dispersion was purchased from Millipore-Sigma. The water was evaporated from the dispersion at room temperature over several days to obtain the 830 EW C2 ionomer in solid form. The solid ionomer was then ground into powder with a mortar and pestle. 3 M Company provided PFSA powders of the C4 (at 725 EW, 790 EW, and 910 EW) and 940 EW LSC, and the sulfonyl fluoride nonionic precursor of 800 EW C4. Aquivion E87–05S (extruded 870 EW C2) membrane was purchased from fuel cell store. All other reagents and solvents were purchased from Millipore-Sigma and used without further purification.

### Dispersion Preparation

PFSA dispersions were prepared according to the method described by Novy et al.^8^ using nPrOH-water, iPrOH-water, and EtOH-water binary solvent compositions. Note that the alcohol or water concentrations reported throughout the present work refer to the relative concentrations of each component in the solvent alone. All measurements were performed on the dispersions exactly 2 days after preparation.

### Rheology and Viscosity Measurements

Shear-dependent rheological measurements were performed on a TA Instruments Discovery HR-30 Rheometer and ARES-G2 Rheometer. The temperature was set at 20 °C for all measurements and controlled within ±0.1 °C by a Peltier plate. The geometry was a 60 mm diameter, 1° cone-and-plate with 25 μm gap. A solvent trap containing a binary alcohol–water mixture matching that of the dispersion solvent was used to minimize solvent evaporation. The PFSA dispersions were equilibrated at 20 °C for 1 min, presheared at 0.1 s^–1^ for 1 min to eliminate any microstructural effects from loading the dispersion into the rheometer and then allowed to relax for another minute. Next, the shear-dependent viscosity, η(γ̇) , was measured from 0.1 to 1000 s^–1^. To obtain zero-shear viscosities and terminal relaxation times, the shear-dependent viscosity data were fit with the Cross model described by eq 1, where γ̇ is the shear rate, η_0_ is the zero-shear viscosity, λ is the terminal relaxation time, and *n* describes the rate of shear thinning.

$$\eta \left(\mathrm{\gamma ̇}\right)=\frac{\eta_{0}}{{1+(\lambda \mathrm{\gamma ̇})}^{n}}$$ 1

Shear-independent viscosity measurements were performed using an Ubbelohde viscometer immersed in a temperature-controlled (±1 °C) water bath. Dispersions were thermally equilibrated in the viscometer for 15 min before the viscosity was measured. The viscometer was cleaned between samples by rinsing three times with acetone, then drying under a stream of air. The reduced viscosity, η_red_, was calculated using eq 2, where *t* and *t*_0_ are the flow times of the ionomer dispersion and pure solvent in the Ubbelohde viscometer, respectively, and C_ionomer_ is the PFSA concentration in units of weight percent.

2 $$\eta_{red}=\frac{t-t_{0}}{t_{0}}\times \frac{1}{C_{ionomer}}$$

### Solubility Parameter Analysis

Pellets of the nonionic sulfonyl fluoride precursor of 800 EW C4 were placed between two 50 μm-thick Kapton films and melt pressed at 250 °C at 6 kPa for 5 min to produce films with thickness >0.1 mm. The sulfonyl fluoride SO_2_F precursor films were hydrolyzed to the ionic SO_3_H form by refluxing in 70 °C 1 M NaOH, then in 70 °C 8 M HNO_3_, for 2 h each. After refluxing in each reagent, excess base or acid was removed from the films by stirring in 70 °C deionized (DI) water for 24 h. The extent of hydrolysis and ion exchange was checked by measuring the highest thermomechanical transition temperature (i.e., *T*_α_)^41–44^ with a TA Instruments Q-800 Dynamic Mechanical Analyzer (DMA). If necessary, the process of refluxing in 70 °C 8 M HNO_3_, then stirring in 70 °C DI water was repeated until only the H^+^-form *T*_α_ was observed by DMA.^42^ Extruded 870 EW C2 membranes were stirred in 70 °C 8 M HNO_3_ for 24 h, then in 70 °C DI water for 24 h.

Samples weighing 100 ± 5 mg were cut from the hydrolyzed H^+^-form 870 EW C2 and 800 EW C4 films and dried under vacuum at 70 °C overnight to obtain the dry membrane mass, *m*_d_. Each sample was placed in a six-dram vial filled with 19 mL of swelling solvent and placed on a roller table for 3 days. The solvent-swollen membrane mass, *m*_s_, was obtained after removing each sample from the vial and gently blotting all solvent remaining on the surface using a KimWipe. The percent solvent uptake was calculated using $100\times \frac{m_{s}-m_{d}}{m_{d}}$ . Then, the backbone and side chain solubility parameters, δ_bb_ and δ_sc_, respectively, were determined using the method developed by Yeo^45^ as shown in Figure S1 in the Supporting Information.

As nonionic sulfonyl fluoride precursors or extruded hydrolyzed membranes were not available for all ionomer chemical structures used in this study, δ_bb_ was extrapolated for the 830 EW C2, 725 EW C4, 910 EW C4, and 940 EW LSC ionomers. As shown in Figure 2a, δ_bb_ values from the C2 and C4 hydrolyzed membranes, Yeo’s values for Nafion,^45,46^ and δ of PTFE^47^ were plotted as a function of n^–1^, where n is the average number of TFE repeat units between side chains. A linear fit to the data was used to extrapolate the δ_bb_ values of the 830 EW C2, 725 EW C4, 910 EW C4, and 940 EW LSC ionomers using the n values reported in Table 1. No clear trend could be observed in a plot of δ_sc_ against ionomer side chain content, as shown in Figure 2b, so it was assumed that δ_sc_ was not significantly affected by side chain content in the range studied here. The δ_sc_ values obtained for the 870 EW C2 and 800 EW C4 membranes, along with δ_sc_ of 1100 EW Nafion from Yeo’s work,^45,46^ were thus assigned to the δ_sc_ values for the 830 EW C2, 725 EW C4, 910 EW C4, and 940 EW LSC ionomers according to side chain length. The assignments of δ_bb_ and δ_sc_ to the five ionomer chemical structures are summarized in Table 1. The solvent-backbone and solvent-side chain interaction parameters, χ_sbb_ and χ_ssc_, respectively, were calculated using the values in Table 1 and eq 3, where δ_polymer_ = δ_bb_ or δ_sc_, *V*_M_ is the solvent molar volume, *R* is the gas constant, and *T* is temperature.

3 $$\chi =\frac{V_{M}}{RT}{\left(\delta_{solvent}-\delta_{polymer}\right)}^{2}+0.34$$

**Figure 2:**
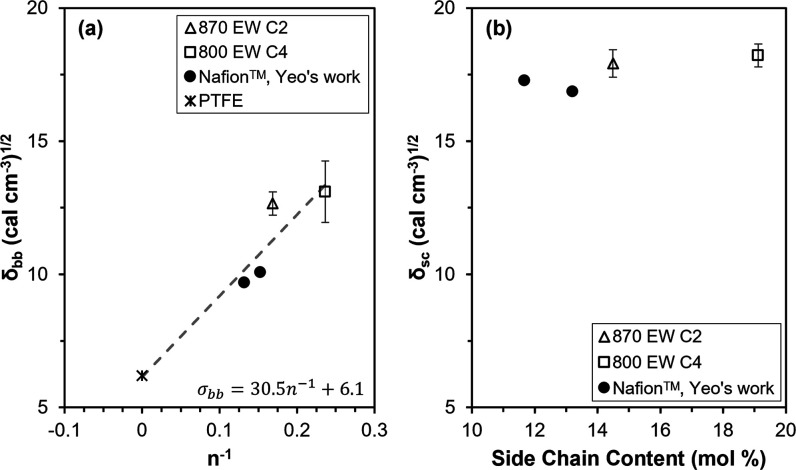
Solubility parameters of the 870 EW C2 and 800 EW C4 ionomers determined by the present study and 1100 EW and 1200 EW LSC (Nafion) determined by Yeo.^45^ (a) Backbone solubility parameters, δ_bb_, as a function of the inverse of the average number of TFE repeat units between side chains, *n* ^–1^, where the dashed line is a linear fit to the data. (b) Side chain solubility parameters, δ_ssc_, as a function of PFSA side chain content.

**Table 1:** PFSA n Values (Average Number of Tetrafluoroethylene Repeat Units Between Side Chains) and Backbone and Side Chain Solubility Parameters, δ_bb_ and δ_sc_, Respectively, Used to Calculate PFSA-Solvent Interactions

Ionomer	n	δ_bb_ (cal cm^–3^)^1/2^	δ_sc_ (cal cm^–3^)^1/2^
830 EW C2	5.5	12 ± 2	17.9 ± 0.5
725 EW C4	3.5	14 ± 2	18.2 ± 0.4
790 EW C4	4.1	13 ± 1	18.2 ± 0.4
910 EW C4	5.3	12 ± 2	18.2 ± 0.4
940 EW LSC	5.0	11 ± 2	16.8 (ref 45)

The binary alcohol–water solvent values, δ_solvent_, at different compositions were calculated using a volume-weighted average of the δ values of the pure alcohol and water. The solvent molar volumes in eq 3 were adjusted to account for excess molar volume associated with mixing.^48,49^

### Small-Angle X-ray Scattering Measurements

Temperature-dependent SAXS measurements were performed at beamline 12-ID-B at the Advanced Photon Source at Argonne National Laboratory (Lemont, Illinois, USA) under General User Proposal No. 68110. To prevent evaporation, PFSA dispersions and solvent blanks were sealed in quartz capillaries (Hampton Research, 1.0 mm O.D.) using epoxy, then mounted in a Linkam THMS600 hot stage with Kapton windows. The temperature was ramped at 10 °C/min from 30 to 80 °C in 10 °C increments, then from 80 to 30 °C. The dispersions were equilibrated at each temperature for 5 min. Two-dimensional SAXS patterns were collected once per minute during the entire heating protocol, using an exposure time of 0.5 s. The incident energy was 13.3 keV, corresponding to X-rays with a wavelength of 0.0932 nm. The detector was a Pilatus 2 M with 0.172 mm pixel size and sample-to-detector distance of 1989 mm. The q-range was calibrated with a silver behenate standard. The SAXS patterns were corrected for sample thickness, transmission, background, and solvent. Fitting of the SAXS patterns with small-angle scattering models was accomplished with Python code in the Spyder integrated development environment.

## Results and Discussion

### Effect of PFSA Chemical Structure and Solvent Composition on the Zero-Shear Viscosity of PFSA Dispersions

The effect of PFSA concentration and chemical structure on dispersion viscosity was investigated at a constant solvent composition. The zero-shear viscosity, η_0_, as a function of PFSA concentration, C, is shown in Figure 3 for dispersions of five different PFSA ionomers in 50 wt % nPrOH (balance water). The data indicate a power law relationship between C and η_0_. Between 10 and 25 wt % PFSA of C4- or LSC-type chemical structure, the η_0_-C power law scaling exponent is close to 3.75, as depicted by the dashed lines in Figure 3a. A scaling exponent of 3.75 has been observed in neutral polymer solutions in good solvent and polyelectrolyte solutions in high salt conditions, and is generally attributed to the semidilute entangled regime.^50–52^ This scaling exponent has also been documented in other salt-free PFSA dispersions in binary alcohol–water solvents^12,34^ and salt-free solutions of rodlike polyelectrolytes.^53,54^ As PFSAs are known to form rodlike colloidal aggregates in binary alcohol–water solvents,^1–8^ the 3.75 scaling exponent can be attributed to the relatively rigid rodlike aggregate morphology in the semidilute entangled regime. In contrast, the scaling exponent of the C2 dispersions, shown by the dashed line in Figure 3b, is approximately 9 between 15 and 25 wt % PFSA. Comparable scaling exponents (6 to 9) have been observed in associating polymer solutions,^55–57^ where the intensified dependence of viscosity on concentration was attributed to the onset of ionic or hydrophobic associations between chains or aggregates.^56–60^ Additionally, positive deviations in η_0_ from the η_0_ ∝ C^3.75^ scaling relationship can be observed above 25 wt % 725 EW C4, 30 wt % 790 EW C4, 35 wt % 910 EW C4, and 40 wt % 940 EW LSC in Figure 3a, similar to the findings of Gupit et al. and Khandavalli et al. for other PFSA dispersions.^12,34^ The observed deviations from the η_0_ ∝ C^3.75^ scaling relationship suggest that, at a certain PFSA concentration, there is a transition between the semidilute entangled regime to a regime where interaggregate associations have a significant contribution to η_0_. Furthermore, the PFSA concentration corresponding to the transition between semidilute entangled and associating regimes appears to depend on the PFSA side chain chemical structure and side chain content.

**Figure 3:**
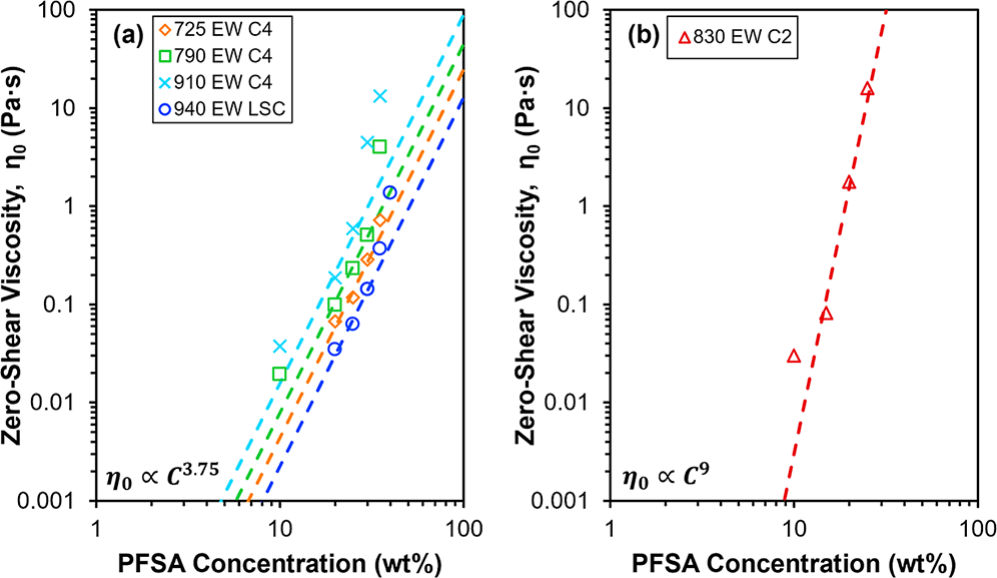
Power law scaling relationships between zero-shear viscosity, η_0_, and PFSA concentration, C, of dispersions in 50 wt % nPrOH (balance water) for (a) 725 EW C4, 790 EW C4, 910 EW C4, and 940 EW LSC, where the dashed lines represent a scaling exponent of 3.75 and (b) 830 EW C2, where the dashed line represents a scaling exponent of 9.

Figure 4a shows the relationship between η_0_ and alcohol concentration in the solvent (balance water), [alcohol], as a function of PFSA chemical structure at a fixed PFSA concentration of 25 wt %. It can be seen that η_0_ increases with increasing [alcohol] for all five PFSA chemical structures and three alcohol–water solvent systems. Similar increases in dispersion viscosity with increasing [alcohol] have been found for 790 EW C2 dispersions in diacetone alcohol–water and 725 EW C4 in iPrOH-water.^10,12^ A sharp, near 10-fold increase in η_0_ can be observed in Figure 4 above a certain critical [alcohol] for the series of three C4 ionomer dispersions in nPrOH-water and 790 EW C4 in iPrOH-water. The critical [alcohol] appears to depend on the side chain content, shifting from about 70 wt % to 50 wt % nPrOH as the side chain content increases from 910 EW to 725 EW (Figure 4a). The critical [alcohol] also changes with solvent composition, occurring at about 55 wt % iPrOH and about 65 wt % nPrOH in dispersions of 790 EW C4 (Figure 4b). At [alcohol] smaller than the critical [alcohol], η_0_ is weakly dependent on solvent composition. In contrast, η_0_ increases by orders of magnitude at [alcohol] higher than the critical [alcohol]. In fact, at alcohol fractions higher than those plotted in Figure 4, η_0_ could not be determined due to gelation of the dispersions. Furthermore, the sharp changes in η_0_ are observed to occur when [alcohol] changes by just 5 wt %. Similar regimes of a weak and strong dependence of viscosity on solvent composition can be observed in the work of Khandavalli et al. on 725 EW C4 dispersions in iPrOH-water,^12^ Acheampong et al. on C2 and LSC dispersions in nPrOH-water,^61^ and Lundberg and Makowski on sulfonated polystyrene (SPS) dispersions in alcohol-xylene.^62^ These studies attributed viscosity increases to the onset of ionic associations between ionomer aggregates or chains. The following section will demonstrate that η_0_ is primarily related to the aggregate surface area at [alcohol] below the critical [alcohol] and strongly affected by interaggregate associations at [alcohol] above the critical [alcohol].

**Figure 4:**
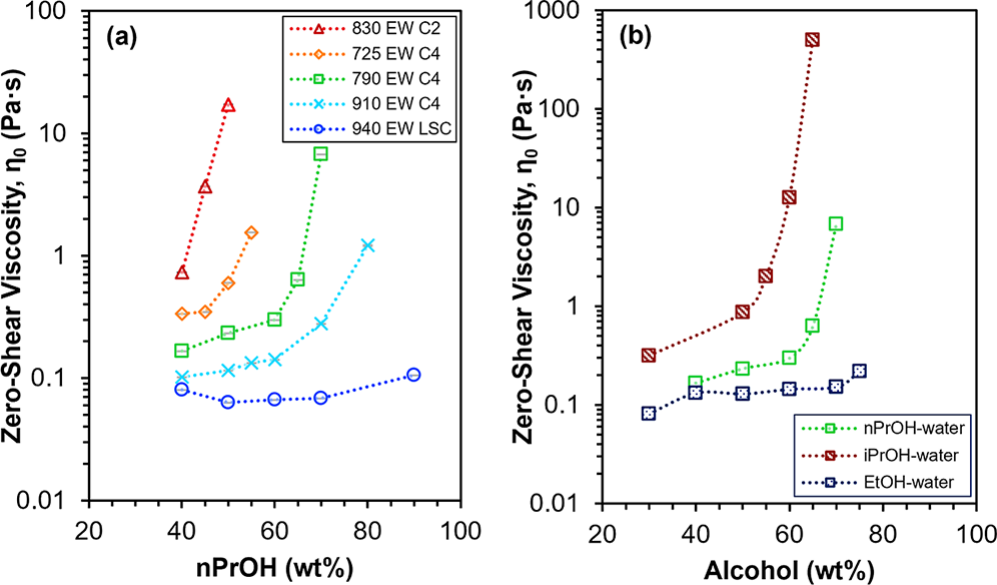
(a) Zero-shear viscosities at 20 °C of 830 EW C2, 725 EW C4, 790 EW C4, 910 EW C4, and 940 EW LSC dispersions as a function of nPrOH concentration in the solvent (balance water) at 25 wt % PFSA. (b) Zero-shear viscosities at 20 °C of 25 wt % 790 EW C4 dispersions in three different binary alcohol–water solvent systems, with nPrOH, iPrOH, or EtOH as the alcohol. Dotted lines are for visualization purposes.

### Relationship between Aggregate Morphology and PFSA Dispersion Viscosity

For PFSA dispersions in the semidilute regime, the increase in η_0_ with increasing [alcohol] may be associated with a change in aggregate morphology. Novy et al. previously demonstrated that the aggregate surface area normalized by side chain content, σ, increases with increasing [alcohol].^8^ The value σ is equal to 2 *V*/*R*, where V is the aggregate volume normalized by PFSA EW and *R* is the aggregate radius obtained from SAXS measurements of PFSA dispersions. The value σ is a measurement of the local morphology of PFSA aggregates, where local refers to length scales less than the average interaggregate spacing. Novy et al. reported an average PFSA interaggregate spacing between 4 and 7 nm.^8^ As viscosity is proportional to the friction between aggregates or polymer chains, it is reasonable to expect that viscosity increases with increasing σ. This is demonstrated by a plot of η_0_ as a function of σ of 25 wt % 790 EW C4 in different binary alcohol–water solvent systems, shown in Figure 5. The dashed lines in Figure 5 correspond to empirical power law fits to the data for σ < 1 nm^2^. A strong dependence of η_0_ on σ can be clearly observed for all systems, suggesting that aggregate morphology as a function of solvent composition can influence dispersion viscosity. However, significant differences in η_0_ can be observed at similar σ (Figure 5). Furthermore, positive deviations from the empirical power law relationship between η_0_ and σ can be observed for dispersions where [alcohol] is greater than the critical [alcohol]. These observations suggest that η_0_ may be influenced by factors in addition to σ, especially above the critical [alcohol].

**Figure 5:**
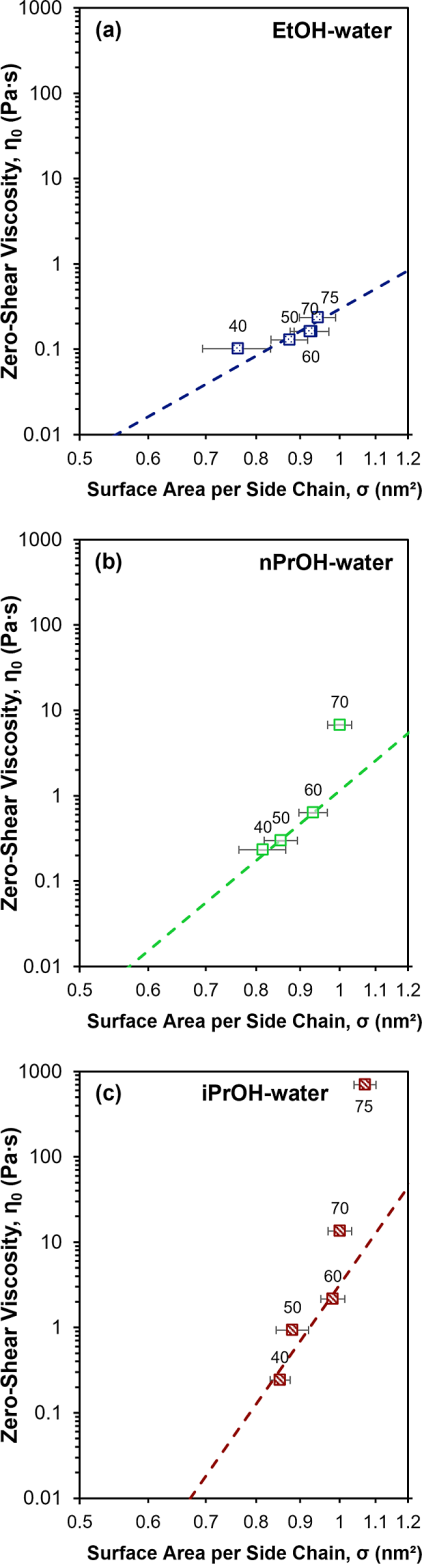
Zero-shear viscosity, η_0_, as a function of the aggregate surface area per side chain, σ, of 25 wt % 790 EW C4 dispersions in binary alcohol–water solvents of (a) EtOH-water, (b) nPrOH-water, and (c) iPrOH-water. The dotted lines represent an empirical power law fit to the data for σ < 1 nm^2^. The label on each data point represents the alcohol composition (balance water) in weight percent in the binary alcohol–water solvent system.

One additional contribution to η_0_ may be interaggregate ionic associations. For example, ionic associations have been attributed to sharp increases in the viscosity of sulfonated ionomer solutions and dispersions.^12,17,62–67^ Viscosity, electron spin resonance, and fluorometry measurements have shown that the addition of small amounts of polar cosolvent can dissipate the ionic interactions through preferential solvation of the sulfonate groups by the polar cosolvent.^62,67–69^ Lundberg and Makowski observed a significant decrease in the reduced viscosity of SPS solutions in binary alcohol-xylene solvent systems with just a small increase in alcohol fraction (a change of about 20 mmol alcohol per 100 mL solvent). The magnitude of the change in reduced viscosity was observed to lessen as the polarity of the alcohol increased. Lundberg and Makowski attributed this trend to improved compatibility between the sulfonate groups and relatively more polar alcohols.^62^ These studies suggest that, in ionomer solutions or dispersions in binary solvent systems, the more polar cosolvent may preferentially solvate the ionic groups, leading to a decrease in ionic associations and decrease in viscosity. In the case of the PFSA dispersions in the present study, the sulfonate groups may be preferentially solvated by water in the binary alcohol–water solvent^10,45,64,70^ leading to fewer or weaker ionic associations at lower alcohol fractions in the semidilute regime.

It should be noted that ionic associations may not be completely dissipated at lower alcohol fractions in semidilute PFSA dispersions. It is expected that ionic associations will increase with increasing PFSA side chain content, i.e., decreasing EW. For example, η_0_ increases in the order 910 EW < 790 EW < 725 EW across a range of nPrOH-water solvent compositions (Figure 4a). Similar increases in viscosity with decreasing EW were observed in 720 and 790 EW C2 dispersions in nPrOH-water solvent.^61^ The near 10-fold increases in η_0_ observed above the critical [alcohol] (Figure 4) may be attributed in part to a significant increase in the magnitude or amount of interaggregate ionic associations due to decreasing preferential solvation of the ionic groups as the water fraction decreases. Together with the η_0_-C scaling relationships for different PFSA chemical structures (Figure 3), the sharp increases in η_0_ above the critical [alcohol] further suggest that an associating regime exists above the semidilute regime. The PFSA concentration corresponding to the transition between the semidilute and associating regimes appears to be dependent on both PFSA chemical structure and solvent composition. Thus, the onset of strong interaggregate ionic associations is affected by ionomer-solvent interactions.

### Ionomer–Solvent Interactions

Interactions between the solvent and PFSA can be quantified using solubility parameters to further elucidate the connection between aggregate morphology, interaggregate associations, and η_0_. The first reported solubility parameter study on Nafion by Yeo established two separate solubility parameters corresponding to the hydrophobic backbone and hydrophilic side chain, δ_bb_ and δ_sc_.^46^ Using Yeo’s methodology for measuring δ_bb_ and δ_sc_ of Nafion, these quantities were determined for the five different PFSA chemical structures used in the present study and are summarized in Table 1. To analyze the ionomer-solvent interactions in detail, the solvent-backbone and solvent-side chain interaction parameters, χ_sbb_ and χ_ssc_, respectively, were calculated as a function of [alcohol] in the solvent using eq 3, where δ_bb_ and δ_sc_ were substituted for δ_polymer_, respectively. In Figure 6, χ_sbb_ and χ_ssc_ of the 830 EW C2, 725 EW C4, 790 EW C4, 910 EW C4, and 940 EW LSC ionomers are plotted as a function of [nPrOH] in the solvent (balance water). It can be seen that χ_sbb_ and χ_ssc_ are nonmonotonic functions of [nPrOH] in the solvent, where χ_sbb_ generally decreases and χ_ssc_ generally increases with increasing [nPrOH]. These trends indicate that solvent-backbone interactions become more favorable and solvent-side chain interactions become less favorable as [nPrOH] in the solvent increases.

**Figure 6:**
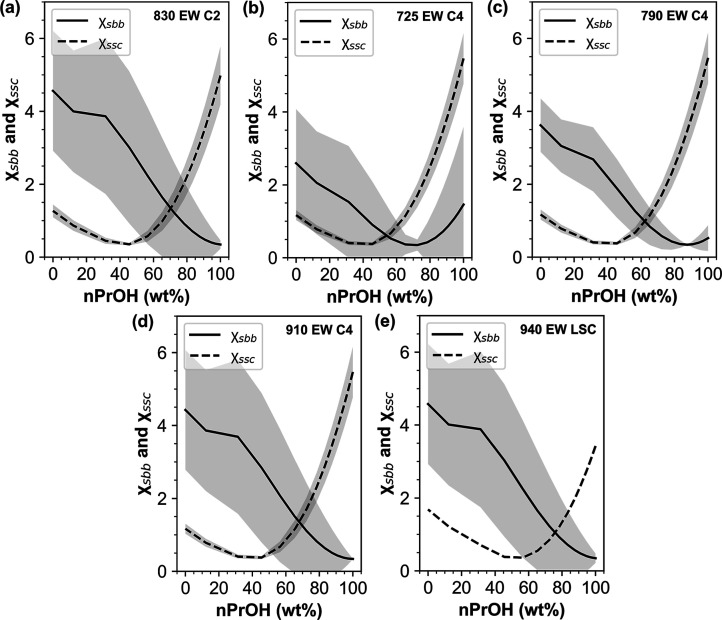
Solvent-backbone and solvent-side chain interaction parameters, χ_sbb_ (solid line) and χ_ssc_ (dashed line), respectively, as a function of [nPrOH] (balance water) in nPrOH-water solvent for (a) 830 EW C2, (b) 725 EW C4, (c) 790 EW C4, (d) 910 EW C4, and (e) 940 EW LSC. Gray shaded regions correspond to the error associated with the calculations determined through standard error propagation calculations.

The decrease in χ_sbb_ with increasing [nPrOH] appears to be qualitatively related to the aggregate morphologies observed by dispersion SAXS by the present authors. It was observed that σ increased with increasing [alcohol] (Figure 5), consistent with increasing favorability of interactions between the solvent and the hydrophobic component of the aggregates.^8^ This observation of the relationship between χ_sbb_ and aggregate morphology is also in agreement with many previous studies and simulations of PFSA dispersions in alcohol–water solvent systems, which suggest that solvation of the PFSA aggregates improves as [alcohol] increases.^8–11,18,71–77^ An increase in σ as χ_sbb_ decreases is consistent with an increase in exposure of the hydrophobic component of the PFSA aggregates to the solvent.^8^ Considering that PFSA aggregates are understood to have a generally rodlike morphology, as demonstrated by previous SAS studies,^3,4,15,17,64,78^ increasing σ may be indicative of an increase in the rodlike character of the aggregates in the semidilute and concentrated regimes over length scales smaller than the average interaggregate spacing. This is in agreement with experimental and modeling studies which suggested that increases in [alcohol] were associated with increases in the length of rodlike PFSA aggregates.^2,13,73^ Furthermore, significant increases in the viscosity of solutions and suspensions of anisotropic particles^79^ and, in the case of cylindrical particles, with a distinct aspect ratio,^80,81^ have been well documented. Thus, increases in η_0_ attributed to increases in σ (Figure 5) appear to be primarily related to an increase in rodlike character of the aggregates that occurs as solvation of the hydrophobic component of the PFSA aggregates improves, i.e., χ_sbb_ decreases. The increase of σ and η_0_ with decreasing χ_sbb_ is also supported by the work of Madsen et al.,^82^ which observed that the intrinsic viscosities of polymer solutions in binary solvent systems reached a maximum when the difference between the solubility parameters of the polymer and binary solvent was the smallest. The observed increases in intrinsic viscosity were attributed to extension of the polymer chains as the compatibility between the solvent and polymer improved.

Figure 6 additionally shows that χ_sbb_ and χ_ssc_ cross over at a certain [nPrOH], [alcohol]_χ_, which suggests the existence of two regimes defined by (I) favorable χ_ssc_ below [alcohol]_χ_ (χ_sbb_ > χ_ssc_) and (II) favorable χ_sbb_ interactions above [alcohol]_χ_ (χ_sbb_ < χ_ssc_). The two regimes above and below [alcohol]_χ_ are reminiscent of the two regimes above and below the critical [alcohol], [alcohol]_η_, observed in the η_0_ data of 25 wt % PFSA dispersions of 725 EW C4, 790 EW C4, and 910 EW C4 as a function of [nPrOH] (Figure 4a). The weak dependence of η_0_ on [nPrOH] in the regime below [alcohol]_η_ was attributed primarily to the influence of σ and thus assigned to the semidilute regime. The much stronger dependence of η_0_ on [nPrOH] above [alcohol]_η_ was attributed to the contribution of both σ and interaggregate ionic associations to η_0_ and thus defined as the associating regime. A comparison between [alcohol]_χ_ and [alcohol]_η_ is made in Figure 7, where the χ_sbb_ and χ_ssc_ curves are overlaid with the η_0_ data of 25 wt % C4 PFSA dispersions as a function of [nPrOH] or [iPrOH]. Strikingly, the value of [alcohol]_χ_ appears to coincide with the value of [alcohol]_η_ within ±5 wt % alcohol, occurring at ca. 55 wt % nPrOH for 725 EW C4, ca. 65 wt % nPrOH for 790 EW C4, and ca. 70 wt % nPrOH for 910 EW C4, and at ca. 55 wt % iPrOH for 790 EW C4 in iPrOH-water.

**Figure 7:**
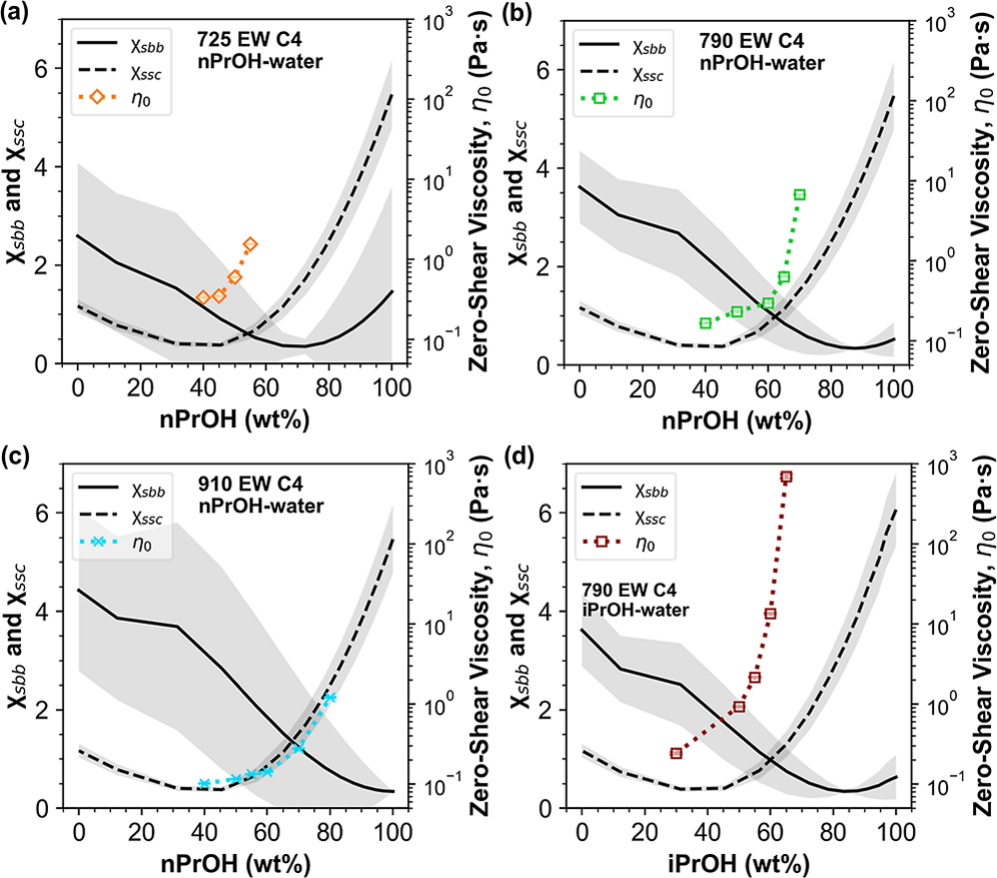
Solvent-backbone and solvent-side chain interaction parameters, χ_sbb_ (solid line) and χ_ssc_ (dashed line), as a function of [alcohol] (balance water) calculated for (a) 725 EW C4, (b) 790 EW C4, and (c) 910 EW C4 in nPrOH and (d) 790 EW C4 in iPrOH. Corresponding zero-shear viscosities, η_0_, of 25 wt % PFSA dispersions of the three ionomers as a function of [nPrOH] are overlaid. The shaded gray regions indicate the error associated with the calculation of χ_sbb_ and χ_ssc_.

However, the analysis of backbone and side chain solubility as a function of [alcohol] may be less applicable to PFSA dispersions at concentrations already in the associating regime. For example, Figure 6a indicates that [nPrOH]_χ_ occurs at ∼70 wt % nPrOH for 830 EW C2. However, Figure 4a shows a strong dependence of η_0_ of 25 wt % 830 EW C2 on [nPrOH] between 40 and 50 wt % nPrOH. At [nPrOH] > 50 wt %, η_0_ of 25 wt % 830 EW C2 dispersions could not be determined due to apparent gelation. Thus, a critical [nPrOH] is not observed at 25 wt % 830 EW C2, as the η_0_ ∝ C scaling relationship shown in Figure 2b indicates that the associating regime of 830 EW C2 in nPrOH-water occurs ≥15 wt % 830 EW C2. This further demonstrates that the transition between semidilute and associating regimes is affected by both PFSA concentration and solvent composition.

As the solubility parameter analysis as a function of [alcohol] appears to be most relevant at semidilute PFSA concentrations, the critical [alcohol] of C2 dispersions is expected to appear at <15 wt % PFSA in the semidilute regime. Acheampong et al. investigated the relative viscosity of 4.5 wt % 790 EW C2 (Aquivion) in the semidilute regime as a function of nPrOH-water solvent composition.^61^ The relative viscosity was observed to increase with increasing [nPrOH] and also demonstrates two regimes of weak and strong dependence of viscosity on solvent composition, consistent with the findings of the present work. The viscosity data of Acheampong et al. indicates that the crossover between weak and strong viscosity dependence occurs at about 65 wt % nPrOH. This [nPrOH] is very near that of the value predicted by our solubility parameter analysis, ∼70 wt % nPrOH (Figure 6a), for 830 EW C2. Note that 830 EW C2 is the same EW within error (±20 EW) to the 790 EW C2 studied by Acheampong et al.^83^ The apparent equivalence of [alcohol]_η_ and [alcohol]_χ_ suggests that [alcohol]_χ_ is an indicator of the transition between the semidilute and associating regimes as a function of solvent composition. This further suggests that interaggregate ionic associations, facilitated by a decrease in relative solvation of side chains, do not strongly contribute to η_0_ until χ_sbb_ < χ_ssc_, i.e., when solvent-side chain interactions become unfavorable relative to solvent-backbone interactions, above [alcohol]_χ_. The onset of interaggregate ionic associations when χ_sbb_ < χ_ssc_ above [alcohol]_χ_ agrees with the study of Lundberg and Makowski regarding ionic associations in Na^+^-form SPS solutions in binary alcohol-xylene solvent systems.^62^

The strength of interaggregate ionic associations in the associating regime may also be interpreted by the magnitude of χ_ssc_ above [alcohol]_χ_. In the solvent composition regime above [alcohol]_χ_, Figure 6 shows that χ_ssc_ decreases in the order C2 ≈ C4 > LSC, which suggests that solvent-side chain interactions, while still comparatively unfavorable above [alcohol]_χ_, are less unfavorable for longer side chain PFSA chemical structures. Thus, the relatively weak ionic associations postulated to occur in 25 wt % 940 EW LSC dispersions may be related to the relative compatibility between the side chain and solvent.

The interaggregate associations of PFSA dispersions can be further investigated through shear-dependent viscosity measurements. Plots of the shear-dependent dispersion viscosities of the five different PFSA chemical structures in 50 wt % nPrOH (balance water) as a function of PFSA concentration are shown in the Supporting Information in Figure S2. Shear thinning, i.e., a decrease in viscosity with increasing shear rate, within the shear rate range of the measurements (∼0.1 to 3000 s^–1^) can be observed in dispersions of ≥25 wt % 725 EW C4, ≥30 wt % 790 EW C4, ≥35 wt % 910 EW C4, and ≥40 wt % 940 EW LSC. For dispersions of the C4 and LSC ionomers, the observation of shear thinning at and above these PFSA concentrations corresponds well to the PFSA concentrations that exhibited a positive deviation from the η_0_ ∝ C^3.75^ scaling relationship (observed in Figure 3a) and that were assigned to the associating regime. For the C2 dispersions, shear thinning can be observed in dispersions of ≥20 wt % 830 EW C2 (Figure S2a), which overlaps with the PFSA concentration regime corresponding to the η_0_ ∝ C^9^ scaling relationship (observed at ≥ 15 wt % 830 EW C2, Figure 3b). Shear thinning of Nafion dispersions has been attributed to the disruption of interaggregate interactions^12,34,84^ and alignment of the aggregates in the direction of shear.^85^ Thus, the observation of shear thinning in dispersions of higher PFSA concentrations further supports the claim that a crossover to an interaggregate associating regime occurs above a certain PFSA concentration, where the crossover concentration is dependent on the PFSA chemical structure, side chain content, and solvent composition. Furthermore, the shear thinning observed in these PFSA dispersions in the associating regime may be related to the disruption of significant interaggregate ionic associations.

It is possible that shear thinning may also be observed in relatively dilute PFSA dispersions, which could support evidence of interaggregate interactions at lower PFSA concentrations. Shear thinning has not been observed in relatively dilute PFSA dispersions (<∼5 wt % PFSA), but at the time of this writing, rheological studies of PFSA dispersions have been limited to shear rates < ∼1000 s^–1^.^12,34,61^ The present work used shear rates up to ∼3000 s^–1^ (Figure S2). However, the onset of shear thinning is expected to occur at higher shear rates as the magnitude of ionic associations decreases. Thus, the contribution of interaggregate associations to viscosity must be relatively small at lower PFSA concentrations.

Interaggregate associations may be evaluated with the Leibler-Rubinstein-Colby (LRC) theory, which was developed to describe the dynamics of entangled reversibly associating polymer networks. The LRC theory predicts that the relaxation time of associating polymer chains, τ_chain_, is related to the number of associating groups, m, per chain and the lifetime of the associations, τ_assoc_, such that τ_chain_ scales as m^2^τ_assoc_.^86^ The terminal relaxation time, τ, of the PFSA dispersions is related to the inverse of the shear rate associated with the onset of shear thinning and can be obtained through fits of viscosity as a function of shear rate to the Cross model (eq 1).^32^ Because shear thinning may only be observed for PFSA dispersions in the associating regime within the range of shear rates used by the present study (∼0.1 to 3000 s^–1^), the terminal relaxation time of the onset of shear thinning may be related to the dynamics of interaggregate ionic associations for dispersions in the associating regime. It must be noted that τ could not be determined for all PFSA concentrations due to (1) the absence of shear thinning over ∼0.1 to 3000 s^–1^ in PFSA dispersions at concentrations below the associating regime and (2) apparent gelation of PFSA dispersions above a certain PFSA concentration that appears to be dependent on PFSA chemical structure. Interaggregate associations may still be present to some degree in dispersions below the associating regime, but were not evidenced by the rheology measurements by the present work. Future rheological studies using shear rates >3000 s^–1^ are required to investigate shear thinning in semidilute PFSA dispersions. Thus, direct comparisons of τ between different PFSA chemical structures at a fixed PFSA concentration cannot be made. However, application of the LRC theory may allow for a qualitative investigation of the contribution of interaggregate associations to η_0_ as a function of PFSA chemical structure and solvent composition.

The characteristic relaxation times, τ, of the PFSA dispersions for which shear thinning was observed are summarized in Tables S1 and S2. The data show near 10-fold increases of τ with either decreasing side chain length or increasing side chain content. Assuming that interaggregate associations are primarily ionic at PFSA concentrations in the associating regime, PFSA side chain content may be related to the number of associating groups, m, of the LRC theory. Assuming a similar lifetime of ionic associations, τ_assoc_, between side chains with the same chemical structure, the increase of τ with increasing side chain content may be related to an increase in the number of interaggregate ionic associations as the number of side chains per aggregate increases. Comparison of the 830 EW C2, 910 EW C4 and 940 EW LSC ionomers with similar side chain content but different side chain chemical structures shows that τ increases as side chain length decreases. Assuming that m is comparable between these three PFSA chemical structures with similar side chain contents, the increase in τ suggests that τ_assoc_ may be longer for shorter side chains, i.e., ionic associations may be stronger between shorter side chains. Simulations of PFSAs with different side chain lengths have indicated that shorter side chains are less flexible and have fewer energetically favorable conformations.^87–90^ Thus, a decrease in side chain flexibility may be associated with the slower dynamics and longer τ observed in PFSA dispersions of shorter side chain PFSA chemical structures. This observation of slower dynamics in dispersions of shorter side chain PFSAs is also supported by the work of Subianto et al.,^91^ which suggested that higher viscosities of 830 EW C2 (Aquivion, Solvay) dispersions compared to 900 EW LSC (Fumion, FuMA-Tech) dispersions were the result of stronger ionic associations between shorter side chains. Furthermore, the dynamics of interaggregate associations interpreted by the LRC theory may help to explain the dependence of the transition between the semidilute and associating regimes on PFSA chemical structure. The transition to the associating regime, related to the onset of positive deviations from the η_0_ ∝ C^3.75^ scaling relationship (Figure 3a), appears to occur at lower PFSA concentrations as the strength or number of interaggregate ionic associations increases.

The presence of two η_0_ regimes above and below [alcohol]_η_ observed in Figure 4a suggests that a critical [water], i.e., concentration of polar cosolvent, is required to destabilize interaggregate ionic associations. In their study of Na^+^-form SPS solutions in binary alcohol-xylene solvent systems, Lundberg and Makowski postulated that dissipation of ionic associations between sulfonate groups could only occur if the ionic associations were sufficiently few or sufficiently weak. If the ionic associations were too strong or too many, no amount of polar cosolvent would be capable of entirely dissipating the ionic associations.^62^ The η_0_ data of dispersions of the three 790 EW C4 ionomers in Figure 4a show that [alcohol]_η_ shifts to lower [nPrOH], i.e., higher [water], as side chain content increases. If the number of ionic associations increases with increasing side chain content as postulated in the paragraph above, a larger fraction of water in the binary solvent system may be required to dissipate a larger number of ionic associations.

Assuming that the lifetime of ionic associations increases in the order LSC < C4 < C2 for different side chain chemical structures at a fixed side chain content, significant ionic associations in the C2 dispersions may be strong enough to persist at [water] as high as 70 wt % (the highest [water] used in the present study), leading to the strongest dependence of η_0_ on solvent composition observed in Figure 4a. Similarly, ionic associations in the LSC dispersions may be sufficiently weak that only a small amount of water (<ca. 10 wt % water) may be necessary for dissipation to occur, resulting in a very weak dependence of η_0_ on solvent composition and the absence of an observable [alcohol]_η_ over the range of 30–90 wt % nPrOH (Figure 4a).

The present analysis has shown that the transition to an associating regime, where ionic associations contribute strongly to dispersion η_0_, depends on PFSA concentration and nPrOH-water solvent composition. To better visualize the effect of dispersion composition on viscosity, η_0_ of dispersions of the five different PFSAs are plotted in ternary diagrams in Figure 8. The zero-shear viscosity of each dispersion is represented by a given color in the colormap in the bottom right of Figure 8, where high viscosities correspond to purple shades and low viscosities correspond to yellow shades. The dispersion compositions where ionic associations are expected to strongly contribute to η_0_ are indicated by the shaded gray region in each ternary diagram. Figure 8 qualitatively shows that the area of the shaded gray regions decreases as side chain length increases or side chain content increases. A smaller shaded gray area reflects a smaller set of dispersion compositions that may result in interaggregate associations.

**Figure 8:**
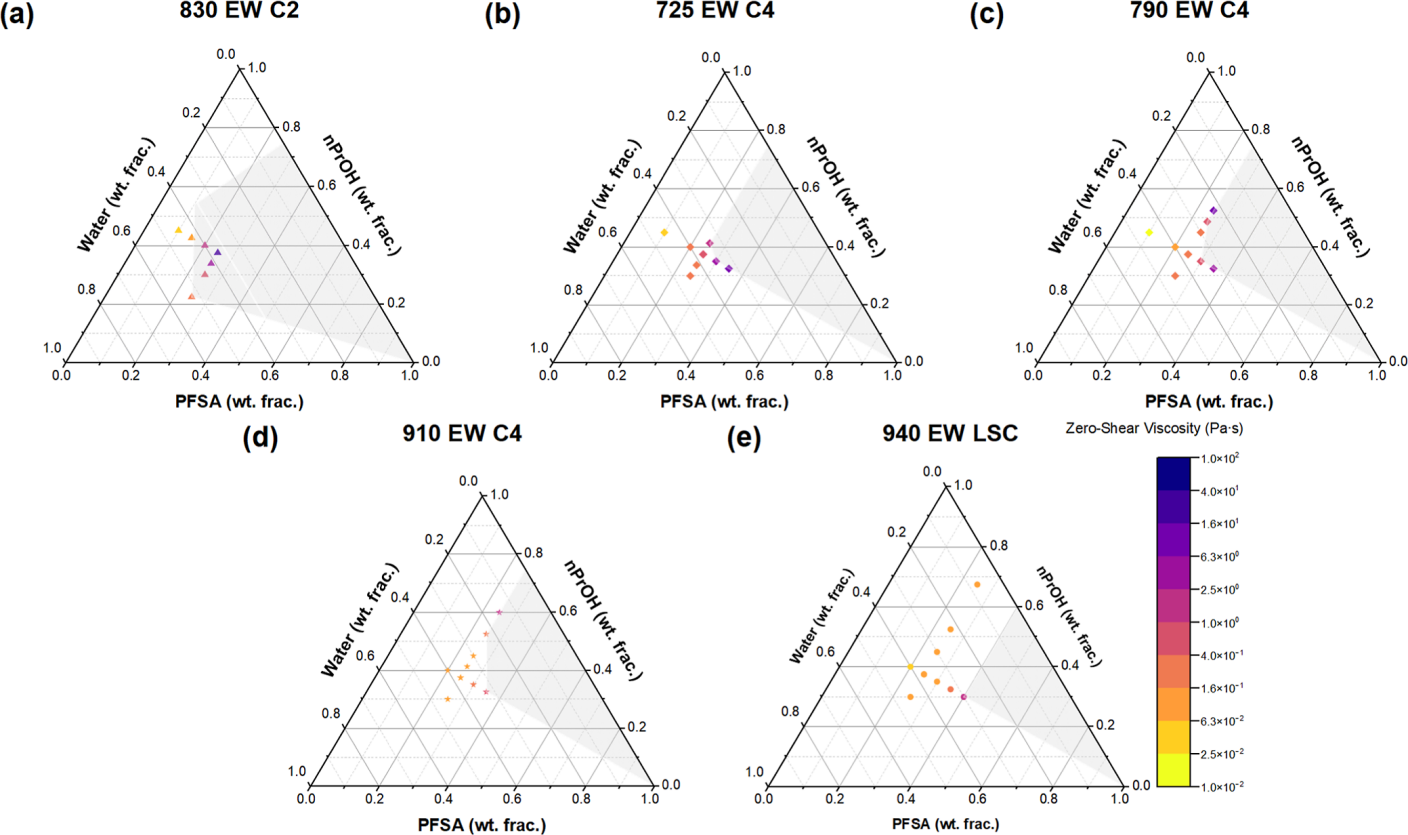
Ternary diagrams of dispersions of (a) 830 EW C2, (b) 725 EW C4, (c) 790 EW C4, (d) 910 EW C4, and (e) 940 EW LSC in nPrOH-water. The dispersion zero-shear viscosity is mapped to a color from the colormap in the bottom right. The gray shaded region in each ternary diagram indicates the associating regime predicted by the present work.

The type of alcohol used in the binary alcohol–water solvent systems in the present study may also affect interaggregate ionic associations. Figure 4b shows that [alcohol]_η_ occurs at ca. 55 wt % alcohol in iPrOH-water and at ca. 65 wt % alcohol in nPrOH-water but appears to be absent from dispersions in EtOH-water up to 75 wt % EtOH. Additionally, at a given alcohol–water solvent composition, η_0_ generally increases in the order of EtOH < nPrOH < iPrOH used as the alcohol in the binary solvent system. The η_0_ trends shown in Figure 4b appear to be similar to those observed by Lundberg and Makowski for Na^+^-form SPS in binary alcohol-xylene solvent systems, where the relative viscosity at a fixed alcohol-xylene composition was shown to decrease as the polarity of the polar cosolvent increased. The authors attributed this decrease in relative viscosity to more pronounced interactions between the polar cosolvent and the sulfonate groups of SPS as the cosolvent polarity increased,^62^ resulting in fewer or weaker ionic associations. Thus, an overall increase in solvent polarity may facilitate the dissipation of interaggregate ionic associations in PFSA dispersions. The relative polarities of the components of the three different binary alcohol–water solvent systems used in the present study increases in the order iPrOH (0.546) < nPrOH (0.617) < EtOH (0.654) < water (1).^92^ Thus, the higher relative polarity of the EtOH-water solvent system, compared to nPrOH-water and iPrOH-water, may decrease the number or strength of interaggregate ionic associations, resulting in the weak dependence of η_0_ on solvent composition and the apparent absence of [alcohol]_η_ from 30 to 75 wt % EtOH (Figure 4b). Comparatively, the strong dependence of η_0_ on solvent composition above [alcohol]_η_ observed in the relatively less polar nPrOH-water and iPrOH-water solvent systems may be associated with relatively more or longer lifetime of ionic associations due to less favorable solvent-sulfonate group interactions. The shift of [alcohol]_η_ to lower [alcohol] as the relative polarity of the alcohol decreases (Figure 4b) suggests a corresponding relative increase in the strength or lifetime of ionic associations. This increase in ionic associations is qualitatively supported by the terminal relaxation times of 25 wt % 790 EW C4 dispersions in different alcohol-water solvent systems shown in Table S2. At a fixed alcohol-water solvent composition, it can be seen that the terminal relaxation time increases by orders of magnitude with decreasing relative polarity of the alcohol.

Ionomer-solvent interaction parameters and thus interaggregate associations are also expected to change with different binary alcohol–water solvent systems. Figure S3 plots χ_ssb_ and χ_ssc_ of the 790 EW C4 ionomer as a function of [alcohol] (balance water), with nPrOH, iPrOH, or EtOH as the alcohol. It can be seen that [alcohol]_χ_ increases in the order iPrOH < nPrOH < EtOH, occurring at ca. 60 wt % iPrOH, ca. 65 wt % nPrOH, and ca. 70 wt % EtOH. Notably, [alcohol]_χ_ ≈60 wt % iPrOH agrees well with [alcohol]_η_ ≈55 wt % iPrOH (Figure 7d). The agreement between [alcohol]_χ_ and [alcohol]_η_ in the nPrOH-water solvent system was discussed earlier in the present section. Above [alcohol]_χ_, χ_ssc_ decreases in the order iPrOH < nPrOH < EtOH (Figure S3). The behavior of [alcohol]_χ_ and χ_ssc_ as a function of binary alcohol–water solvent composition are consistent with the onset and strength of interaggregate ionic associations above [alcohol]_η_. The strength of the ionic associations was suggested to increase as the relative polarity of the binary alcohol–water solvent system decreased, leading to the occurrence of [alcohol]_η_ at lower [alcohol]. The decrease in relative polarity of the binary alcohol–water solvent appears to be related to an increase in χ_ssc_. Thus, the weakest ionic associations postulated to occur in 25 wt % 790 EW C4 dispersions in binary EtOH-water solvents (Supporting Information) may be attributed to the smaller χ_ssc_ above [alcohol]_χ_.

Selective interactions of binary alcohol–water solvent systems with the hydrophobic and hydrophilic components of PFSAs have been qualitatively noted in many studies of PFSA dispersions, catalyst inks, membranes, and thin films.^45,62,70,93–95^ However, the present study appears to be the first to quantify PFSA-solvent interaction parameters as a function of binary alcohol–water solvent composition and identify a regime change between favorable solvent-side chain interactions and favorable solvent-backbone interactions, delineated by [alcohol]_χ_. Applied to nondilute PFSA dispersions, modeling χ_sbb_ and χ_ssc_ as a function of [alcohol] appears to be able to predict the crossover between the semidilute and associating regimes, [alcohol]_χ_, within ±5 wt % alcohol in 25 wt % 725 EW C4, 790 EW C4, and 910 EW C4 dispersions in nPrOH-water and 25 wt % 790 EW C4 dispersions in iPrOH-water. However, [alcohol]_χ_ may have less significance in nondilute dispersions where (I) ionic associations strongly contribute to dispersion η_0_, i.e., 830 EW C2 in nPrOH-water, or where (II) ionic associations are fairly dissipated due to dynamics associated with the PFSA chemical structure (Supporting Information) or relatively favorable solvent-side chain interactions, i.e., 25 wt % 940 EW LSC in nPrOH-water and 25 wt % 790 EW C4 in EtOH-water. The investigation of PFSA aggregate morphologies and dispersion viscosities in the present study has shown that PFSA dispersions are likely subject to a complex interplay of ionomer-solvent and ionomer–ionomer interactions. To achieve a more comprehensive understanding of the influence of parameters such as PFSA chemical structure and solvent composition on PFSA dispersion morphology, evaluation of χ_sbb_ and χ_ssc_ must be considered alongside other factors, including hydrophobic/ionic aggregation and intra/interaggregate interactions.

### Effect of Temperature on Dispersion Morphology and Viscosity

PFSA dispersion morphology in the context of ionomer-solvent interactions was further investigated by varying the temperature of both 1 and 25 wt % dispersions of 790 EW C4 in 50 wt % nPrOH (balance water). The PFSA concentration of 1 wt % was selected for (I) relevance to typical PFSA concentrations in catalyst inks and dispersions to fabricate thin films,^18,96^ (II) comparison to other SAS studies of PFSA dispersion morphology, and (III) comparison to temperature-dependent viscosity measurements of dilute PFSA dispersions to be discussed later in this section.

Dispersion SAXS patterns of both the 1 and 25 wt % PFSA dispersions as a function of temperature from 30 to 80 °C are shown in Figure 9a,b. The low-q upturn observed at *q* < ca. 0.2 nm^–1^ and scattering maximum, *q*_max_, at *q* = ca. 1 nm^–1^ for the 25 wt % PFSA dispersion and at *q* = ca. 0.24 nm^–1^ for the 1 wt % PFSA dispersion are commonly observed in SAS patterns of solutions of charged polymers^97–104^ A shoulder at *q* = ca. 0.7 nm^–1^, occurring at higher q than the primary scattering maximum, *q*_max_, can be observed for the 1 wt % 790 EW C4 dispersion (Figure 9a). This shoulder at q > *q*_max_ has also been observed in < ca. 5 wt % PFSA dispersions and solutions of hydrophobic polyelectrolytes, where it has been attributed to intra-aggregate scattering, i.e., the form factor.^73,105,106^

**Figure 9:**
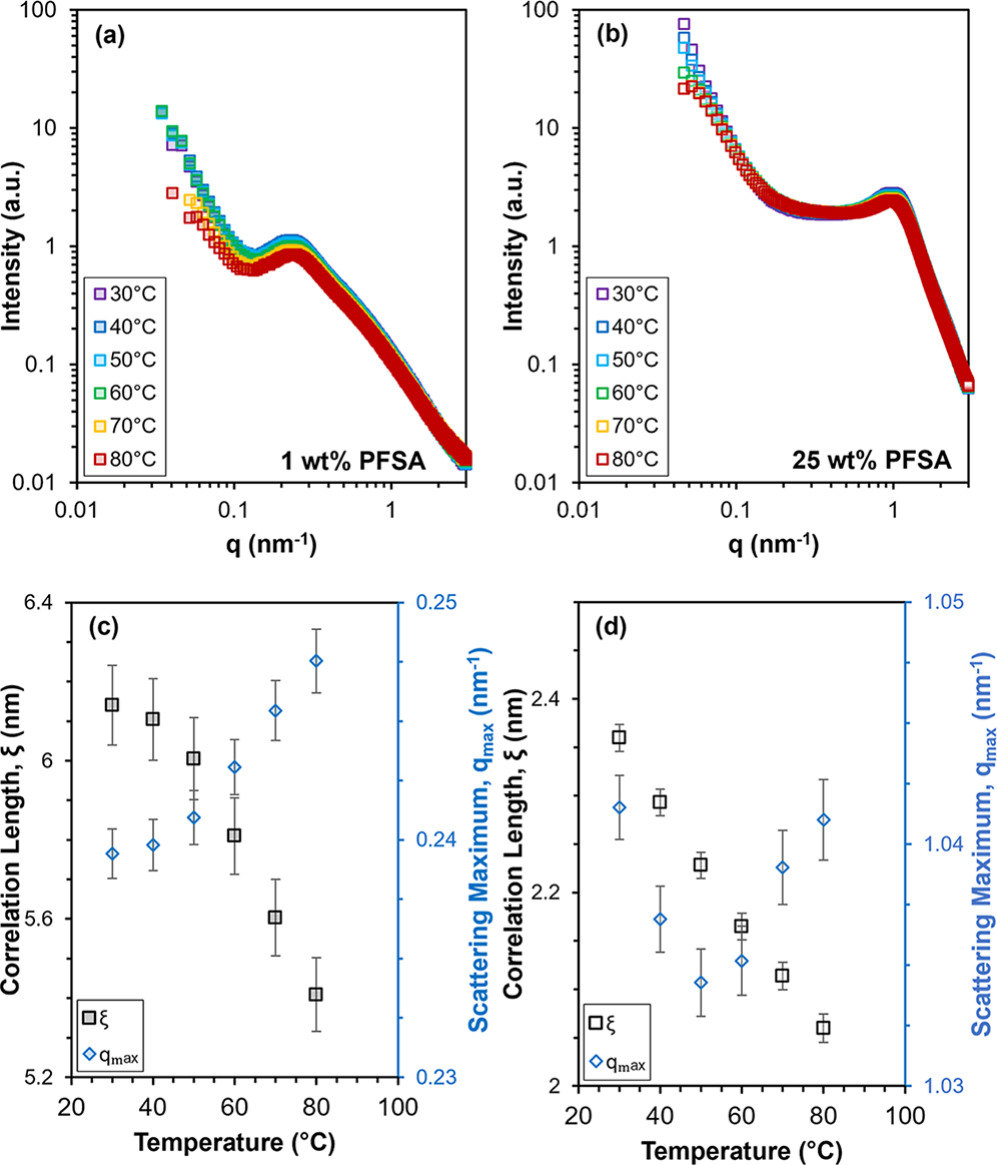
Top row: temperature-dependent dispersion SAXS patterns of 790 EW C4 dispersions in 50 wt % nPrOH at (a) 1 wt % PFSA and (b) 25 wt % PFSA. Each SAXS pattern was obtained after 5 min of equilibration at the corresponding temperature. Bottom row: Correlation length, ξ, and scattering maximum, *q*_max_, of 790 EW C4 in 50 wt % nPrOH as a function of temperature obtained from SAXS patterns of (c) a 1 wt % PFSA dispersion fit with the model of Hammouda and co-workers and (d) a 25 wt % 790 EW C4 PFSA dispersion fit with the Teubner–Strey model.

Figure 9a,b shows that the low-q upturn and *q*_max_ observed in the 25 wt % PFSA and 1 wt % PFSA dispersions, and the additional higher-q shoulder observed only in the 1 wt % PFSA dispersion, decrease slightly in intensity as the dispersion temperature increases. This slight decrease in intensity of the low-q upturn, *q*_max_, and higher-q shoulder is more noticeable in the SAXS patterns of the 1 wt % PFSA dispersion (Figure 9a). This suggests that the PFSA aggregate morphology may change with temperature, where the aggregate morphology of the 1 wt % PFSA dispersion is more sensitive to temperature compared to the 25 wt % PFSA dispersion.

The 25 wt % 790 EW C4 dispersion SAXS patterns (Figure 9b) were fit with the Teubner–Strey model^107^ to quantify the changes in PFSA aggregate morphology with temperature. However, the 1 wt % PFSA dispersion SAXS patterns (Figure 9) could not be fit with the same Teubner–Strey model, as this model cannot accurately recreate the shallower high-q slope at *q* > ca. 0.25 nm^–1^ of the 1 wt % PFSA dispersion SAXS patterns, in comparison to the steeper high-q slope at *q* > ca. 1 nm^–1^ of the 25 wt % PFSA dispersion SAXS patterns. This is likely because the Teubner–Strey model was originally developed for two-phase, bicontinuous systems,^107,108^ and thus may not be applicable to comparatively dilute PFSA dispersions. Instead, the 1 wt % PFSA dispersion SAXS patterns were fit with a model developed by Hammouda and co-workers^109^ to empirically describe the low-q upturn and scattering maximum typically observed in SAS patterns of salt-free solutions of polyelectrolytes. Details of fitting the SAXS patterns with the model of Hammouda et al. are given in the Supporting Information. An example of the fit of the Hammouda et al. model to the dilute PFSA dispersion SAXS patterns is shown in Figure S4 and Table S3. Notably, both the Teubner–Strey model and the model of Hammouda et al. include a fitting parameter describing the length scale of correlations between scattering particles, the correlation length ξ. Smaller values of ξ are associated with lower spatial ordering of aggregates and decreased aggregation.^8^

The ξ and *q*_max_ values of the 25 wt % PFSA dispersion and of the 1 wt % PFSA dispersion are plotted in Figure 9c,d as a function of temperature. It can be observed that *q*_max_ of the 1 wt % PFSA dispersion is overall smaller than that of the 25 wt % PFSA dispersion, which indicates that average interaggregate distances are larger in dilute PFSA dispersions, as expected. The *q*_max_ values of both dispersions changes by *q* < ca. 0.01 nm^–1^ over the entire temperature range, suggesting that temperature does not significantly affect the average distance between aggregates.

In both the 1 and 25 wt % PFSA dispersions, it can be seen that ξ decreases with increasing temperature (Figure 9c,d). As the parameter ξ is inversely proportional to the width of the scattering maximum in both the Teubner–Strey model^107^ and the model of Hammouda et al.,^109^ the decrease in ξ suggests that spatial order in the dispersions decreases as the temperature increases. Additionally, this decrease in ξ appears to occur in both the 1 and 25 wt % PFSA dispersions, although the magnitude of the decrease in ξ is larger for the latter. The decrease in spatial order associated with a decrease in aggregation may indicate an improvement in PFSA-solvent interactions. An improvement in PFSA-solvent interactions with increasing temperature is consistent with the inverse relationship between temperature and χ apparent in eq 3.

The reduced viscosities, η_red_, of dilute, <0.6 wt % 790 EW C4 dispersions in 50 wt % nPrOH are shown in Figure 10 as a function of PFSA concentration and temperature from 23 to 80 °C. The increase in η_red_ with decreasing PFSA concentration at a fixed temperature demonstrates the polyelectrolyte effect, which has been previously reported for other PFSA dispersions in polar solvents.^3,12,16,35^ The polyelectrolyte effect is attributed to chain extension or expansion in solution as the polymer concentration decreases.^97,110,111^ It is thought that charged groups along a chain experience less charge screening by neighboring chains as polymer concentration decreases, leading to an increase in intrachain electrostatic repulsions and consequently extension of the chain. A similar effective increase in the rodlike character of colloidal aggregates is postulated to occur in PFSA dispersions in the dilute regime.^16,35^ It is possible that the conformation of a colloidal aggregate resulting from the aggregation of multiple extended PFSA chains may reflect that of the component chains, to some extent. This suggestion is supported by the work of Lee et al. on dilute Nafion dispersions in 75 wt % MeOH (balance water), which used polarized light scattering to measure an increase in the anisotropy of the rodlike aggregates as the PFSA concentration decreased.^14^

**Figure 10:**
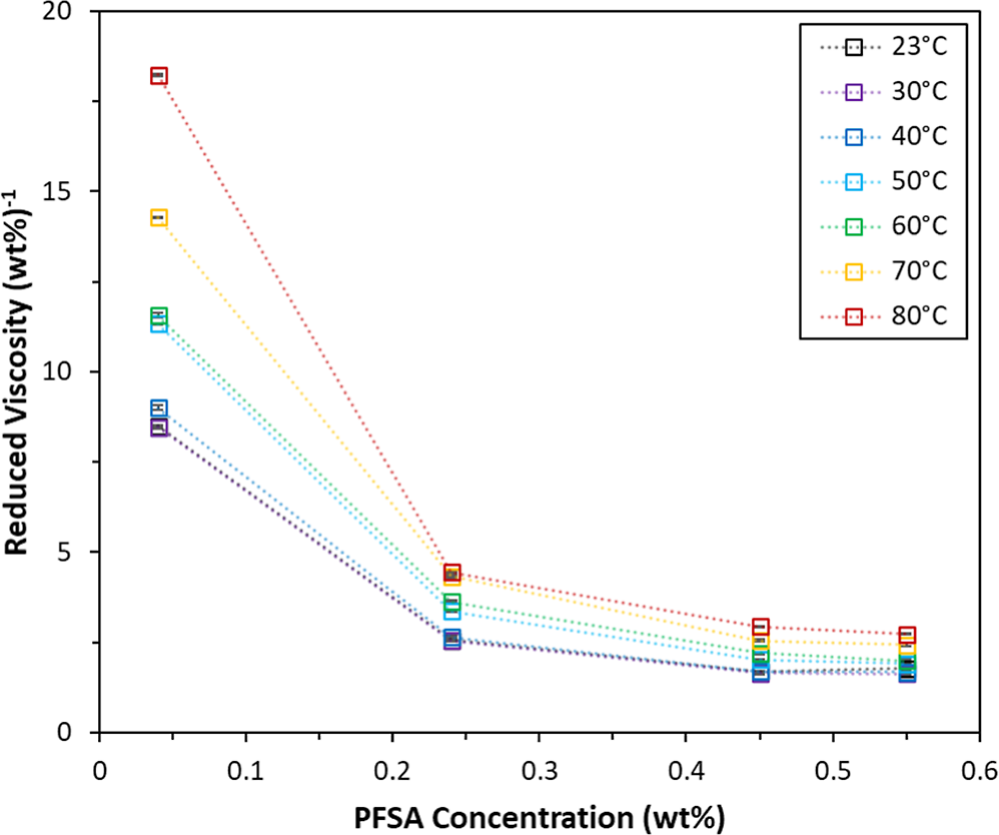
Reduced viscosity of dilute 790 EW C4 dispersions in 50 wt % nPrOH (balance water) as a function of temperature from 23 to 80 °C. The dotted lines are for visualization purposes.

Figure 10 shows that η_red_ increases with increasing temperature for all PFSA concentrations that were examined, suggesting that higher temperatures increase the polyelectrolyte effect. An intensified polyelectrolyte effect at higher temperatures has been observed in solutions of low-charge fraction SPS and attributed to improved solvation and thus larger hydrodynamic volumes of the SPS chains with increasing temperature.^62,112^ Thus, the increase in η_red_ with increasing temperature suggests increasing compatibility between the solvent and PFSA aggregates, in agreement with the observation of reduced aggregation by temperature-dependent dispersion SAXS. The dependence of η_red_ and PFSA aggregation on temperature is consistent with the inverse relationship between χ and temperature described by eq 3. Thus, an increase in the favorability of PFSA-solvent interactions with increasing temperature appears to result in PFSA aggregates with larger hydrodynamic volumes, or possibly increased anisotropy of the rodlike aggregates.

## Conclusions

PFSA-solvent and interaggregate interactions have a fundamental influence on the morphology and viscosity of PFSA dispersions. Dispersion SAXS measurements of nondilute PFSA dispersions in a previous publication by the present authors showed that the morphology of PFSA aggregates depends on the solvent-aggregate interfacial energy. The aggregate surface area normalized by side chain content, σ, was found to increase as the alcohol fraction of the binary alcohol–water solvent increased. Calculation of the solvent-backbone interaction parameter, χ_sbb_, as a function of solvent composition showed that increases in σ were related to improved compatibility between the solvent and the hydrophobic component of the aggregates. This increase in σ was also related to increases in dispersion zero-shear viscosity, η_0_, with increasing [alcohol]. Two regimes of weak and strong dependence of η_0_ on solvent composition were identified to occur below and above a critical [alcohol], respectively, and attributed to regimes of different interaggregate interactions. The strong dependence of η_0_ above the critical [alcohol] was attributed to interaggregate ionic associations that could occur at sufficiently high PFSA concentrations. Interaggregate ionic associations were additionally shown to occur when the solvent-side chain interaction parameter, χ_ssc_, was unfavorable compared to χ_sbb_. The strength of interaggregate ionic associations and therefore the strength of the dependence of η_0_ on solvent composition was shown to increase with increasing χ_ssc_. The existence of two regimes of different interaggregate interactions as a function of PFSA chemical structure and solvent composition was confirmed by the equivalence of the critical [alcohol] observed by η_0_ measurements and [alcohol] above which χ_sbb_ < χ_ssc_. The transition to the associating regime at a fixed, nondilute PFSA concentration was shown to depend on PFSA chemical structure and solvent composition. The calculation of χ_sbb_ and χ_ssc_ was also in agreement with temperature-dependent measurements of dilute PFSA dispersion morphology and viscosity. A decrease in spatial order of PFSA dispersions with increasing temperature was in qualitative agreement with decreases in both χ_sbb_ and χ_ssc_, suggesting that higher temperatures may improve PFSA-solvent interactions. Reduced viscosity measurements of dilute PFSA dispersions demonstrated the polyelectrolyte effect. The polyelectrolyte effect was shown to intensify with increasing temperature, which suggested that improved PFSA-solvent interactions result in an increase of the hydrodynamic volume or rodlike character of the aggregates.

The PFSA-solvent interaction parameters χ_sbb_ and χ_ssc_ may help to inform rational selection of processing parameters for PFSA membranes, thin films, and catalyst inks using binary solvent systems. Provided that the solubility parameters of the PFSA and solvent are known, χ_sbb_ and χ_ssc_ may predict dispersion viscosity behavior and aggregate morphology. For example, higher viscosity dispersions with slower dynamics due to strong ionic associations may require longer thermal treatment times or higher processing temperatures to achieve a level of morphological development in the final film that is sufficient for proton-exchange membrane applications. Additionally, adjustment of the relative solvation of the side chain and backbone through solvent composition for a given PFSA chemical structure may be able to control phase separation of the hydrophilic and hydrophobic phases during film formation.

The findings of this work have demonstrated that PFSA-solvent interactions are an underlying contribution to PFSA colloidal morphology and viscosity behavior. However, this analysis of interaction parameters must be evaluated in addition to other parameters such as Hansen solubility parameters, interfacial energies, and solvent dielectric constants to achieve a better understanding of PFSA dispersion morphology. It must also be noted that PFSA-solvent interaction parameters, which are independent of PFSA concentration and interaggregate interactions, may have different interpretations in PFSA concentration-dependent regimes of different interaggregate interactions, i.e., dilute, semidilute, and associating. For example, dissipation of interaggregate ionic associations in nondilute dispersions appeared to result from relatively favorable χ_ssc_ values. However, relatively favorable χ_ssc_ values may indicate an increase in electrostatic interactions in the dilute regime. PFSA-solvent interaction parameters should be examined in other systems to determine the extent of its interpretation and applicability. Dispersions at dilute PFSA concentrations, high-boiling point solvents, and novel ionomer chemical structures are prime candidates for this analysis to advance the fundamental understanding of PFSA colloidal dispersion morphologies.

## Supplementary Material



## Notes

Additional information on determining PFSA solubility parameters, ionic associations in ionomers, and fitting SAXS patterns of dilute PFSA dispersions (PDF)

## §

Present Address Johnson Matthey Technology Centre, Blounts Court, Sonning Common, Reading, RG4 9NH, United Kingdom

## Abbreviations

EtOH: ethanol
EW: equivalent weight
iPrOH: isopropanol
LRC: Leibler-Rubinstein-Colby
MeOH: methanol
nPrOH: n-propanol
PEM: proton-exchange membrane
PFSA: perfluorosulfonic acid ionomer
SAS: small-angle scattering
SAXS: small-angle X-ray scattering
SPS: sulfonated polystyrene
TFE: tetrafluoroethylene
