# Supplementary material for: Effect of Ionomer–Solvent Interactions in PFSA Dispersions: Dispersion Viscosity
Source: Macromolecules. 2025 Dec 13;58(24):13336–51. doi: 10.1021/acs.macromol.5c00637 (PMC12752697; doi:10.1021/acs.macromol.5c00637)
Supplement: Supplementary file 1 [file ma5c00637_si_001.pdf]

SUPPORTING INFORMATION

EFFECT OF IONOMER-SOLVENT INTERACTIONS IN PFSA DISPERSIONS: DISPERSION VISCOSITY

Melissa Novy,[†,§] Denis Duchesne,[‡,§] Gregg Dahlke,[‡] Lisa P. Chen,[‡] and Robert B. Moore[,†]*

**Email: rbmoore3@vt.edu*

[†] Department of Chemistry, Macromolecules Innovation Institute, Virginia Tech, Blacksburg, Virginia, 24061, United States.

[‡] 3M Advanced Materials Division, 3M Center, Building 280-1W-03, St. Paul, Minnesota 55144, United States.

Present Address

[§] Johnson Matthey Technology Centre, Blounts Court, Sonning Common, Reading, RG4 9NH, United Kingdom.

Backbone and Side Chain Solubility Parameters

The backbone and side chain solubility parameters, δ_{bb} and δ_{sc}, respectively, of hydrolyzed 870 EW C2 and 800 EW C4 membranes were calculated from solvent uptake data using the methodology developed by Yeo.[1] The polymer volume fraction, v, of solvent-swollen membranes was used to calculate the polymer-solvent interaction parameter, χ, using **eq S1**, where $V_{solvent}$ is the molar volume of the swelling solvent, R is the gas constant, T is temperature, ρ is the PFSA density $\approx$ 2.1 g/cm^3, and EW is the equivalent weight of the PFSA. The relationship between χ and the polymer solubility parameter, $\delta_{polymer}$, is demonstrated in **eq S2**, where $\delta_{solvent}$ is the solubility parameter of the solvent and ζ is a constant between 0.1 and 0.4.[1]

$$\ln(1-v)+v+\chi v^2=\frac{2V_{solvent}C}{RT}\left(v^{\frac{1}{2}}-\frac{1}{2}v\right) \qquad (S1)$$

$$\text{where } C=\frac{RT\rho}{2EW}$$

$$\frac{\delta_{solvent}^2}{RT}-\frac{\chi}{V_{solvent}}=\left(\frac{2\delta_{polymer}}{RT}\right)\delta_{solvent}-\frac{\delta_{polymer}^2}{RT}-\frac{\zeta}{V_{solvent}} \qquad (S2)$$

The left side of **eq S2** is plotted as a function of $\delta_{solvent}$ for the 870 EW C2 and 800 EW C4 hydrolyzed membranes in **Figure S1**. Plots of the left side of **eq S2** as a function of the solubility parameter of the swelling solvent, $\delta_{solvent}$ for hydrolyzed membranes of (A) 870 EW C2 and (B) 800 EW C4. The dashed and dotted lines are linear fits to the data, corresponding to swelling of the hydrophobic domains and swelling of the hydrophilic domains by the swelling solvent, respectively.[1] **Equation S2** indicates that the slope of the lines of best fit is equal to $\frac{2\delta_{polymer}}{RT}$. Thus, the slope of the dashed line may be used to obtain δ_{bb} by letting δ_{bb} equal $\delta_{polymer}$ and solving for δ_{bb}. The same approach is applied to the slope of the dotted line to obtain δ_{sc}.

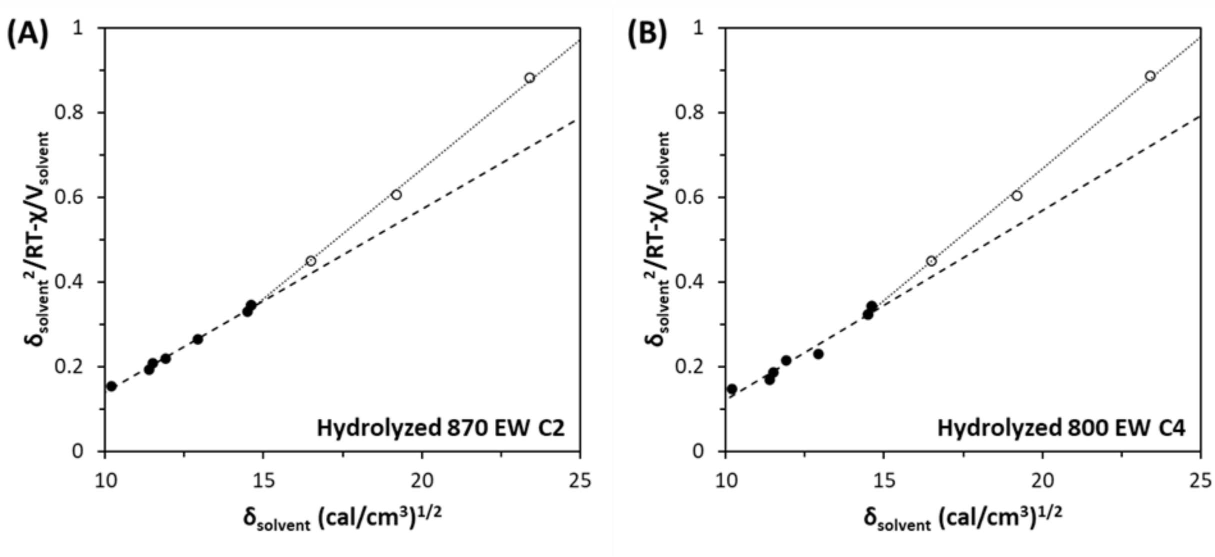

Figure S1. Plots of the left side of eq. S2 as a function of the solubility parameter of the swelling solvent, $\delta_{solvent}$ for hydrolyzed membranes of (A) 870 EW C2 and (B) 800 EW C4. The dashed and dotted lines represent linear fits to the data.

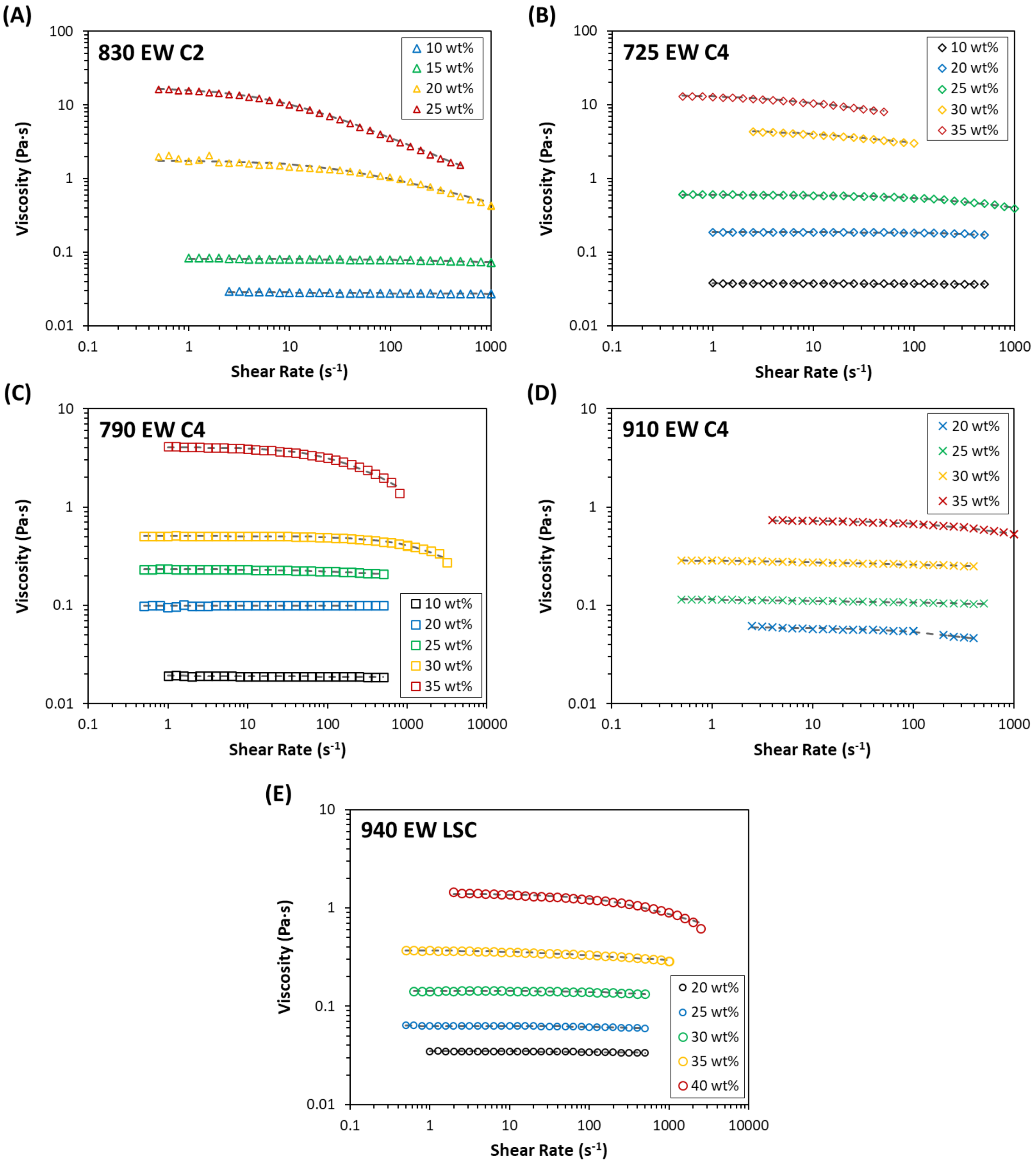

Figure S2. Shear-dependent viscosities of PFSA dispersions in 50 wt% nPrOH (balance water) as a function of PFSA concentration: (A) 830 EW C2, (B) 725 E C4, (C) 790 EW C4, (D) 910 EW C4, and (E) 940 EW LSC. Dashed lines are fits to the Cross model (**eq 1**).

Table S1. Terminal relaxation times of PFSA dispersions in 50 wt% nPrOH (balance water) as a function of PFSA concentration. The relaxation times were obtained by fitting the shear-dependent viscosity data shown in **Figure S2** with the Cross model. The terminal relaxation time is related to the inverse of the shear rate associated with the onset of shear thinning. The symbol * indicates that a terminal relaxation time could not be determined due to the absence of shear thinning over the investigated range of shear rates (~0.1 to 3000 s^{-1}).

PFSA Concentration (wt%)	Terminal Relaxation Time, τ (ms)				
	830 EW C2	725 EW C4	790 EW C4	910 EW C4	940 EW LSC
10	*	*	*		
15	*				
20	10 ± 3	*	*	*	*
25	70 ± 2	*	*	*	*
30		4.1 ± 0.3	0.21 ± 0.01	*	*
35		12.5 ± 0.1	2.3 ± 0.1	0.20 ± 0.01	*
40					0.45 ± 0.02

Table S2. Terminal relaxation times as a function of alcohol-H_2O solvent composition obtained from fitting shear-dependent viscosity data of 25 wt% 790 EW C4 dispersions with the Cross model. The symbol * indicates that a terminal relaxation time could not be determined due to the absence of shear thinning over the investigated range of shear rates (~0.1 to 3000 s^{-1}).

Alcohol (wt%)	Terminal Relaxation Time, τ (ms)		
	nPrOH-H_2O	iPrOH-H_2O	EtOH-H_2O
30		*	*
40	*		*
50	*	*	*
55		*	
60	0.19 ± 0.02	290 ± 70	*
65	0.88 ± 0.01	1900 ± 500	
70	150 ± 10		*
75			*

Effect of Alcohol Type on Ionomer-Solvent Interaction Parameters

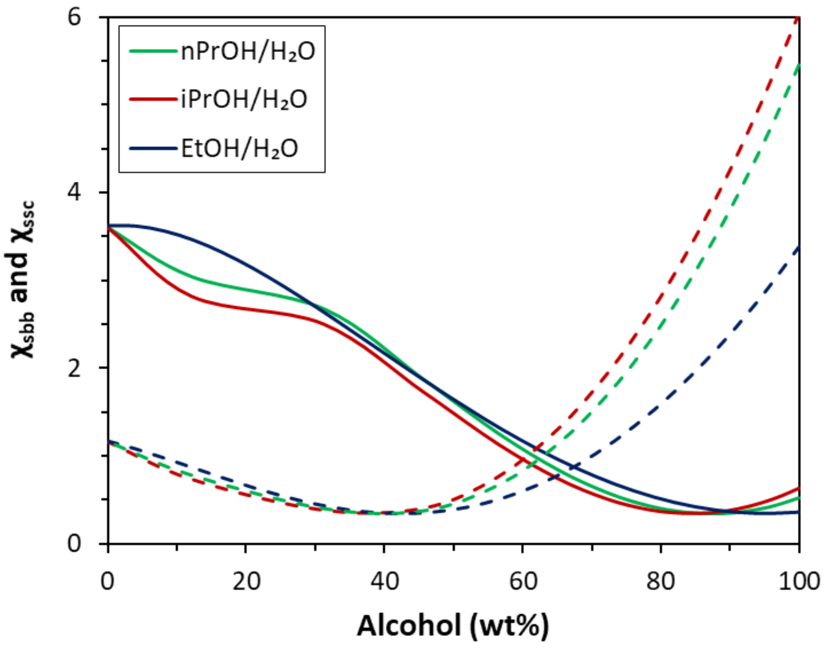

Figure S3. χ_{sbb} (solid lines) and χ_{ssc} (dashed lines) calculated for 790 EW C4 as a function of alcohol concentration (balance H_2O) of three binary alcohol-water solvent systems, with nPrOH (green), iPrOH (red), or EtOH (blue) as the alcohol.

Fitting SAXS Patterns of Dilute PFSA Dispersions

The SAXS patterns of 1 wt% 790 EW C4 dispersions collected at different temperatures (**Figure 9a**) were fit with the model of Hammouda et al.[2] This model is described by **eq S3**, where the first and last terms are a power law representing the low-q upturn. A_{H1} is the scaling factor, D is the power law slope, and B is a constant background arising from incoherent scattering. The scattering maximum is described by the second term in **eq S3**, where A_{H2} is the scaling factor of the second term, q_{max} is the position of the scattering maximum, ξ is the correlation length, and m is the power law slope of the scattering intensity for $q > q_{max}$. The low-q power law, q_{max}, ξ, and BG parameters have the same meanings as in the Teubner-Strey model. Notably, ξ is defined as the length scale

of correlations between scattering particles in both the Teubner-Strey model[3] and the model of Hammouda et al.[2] and is thus related to the degree of local order in the PFSA dispersions. An example of the fit of the Hammouda et al. model to the dilute PFSA dispersion SAXS patterns is shown in **Figure S4** and **Table S3**.

$$I(q) = \frac{A_{H1}}{q^D} + \frac{A_{H2}}{1 + (|q - q_{max}|\xi)^m} + BG \qquad (S3)$$

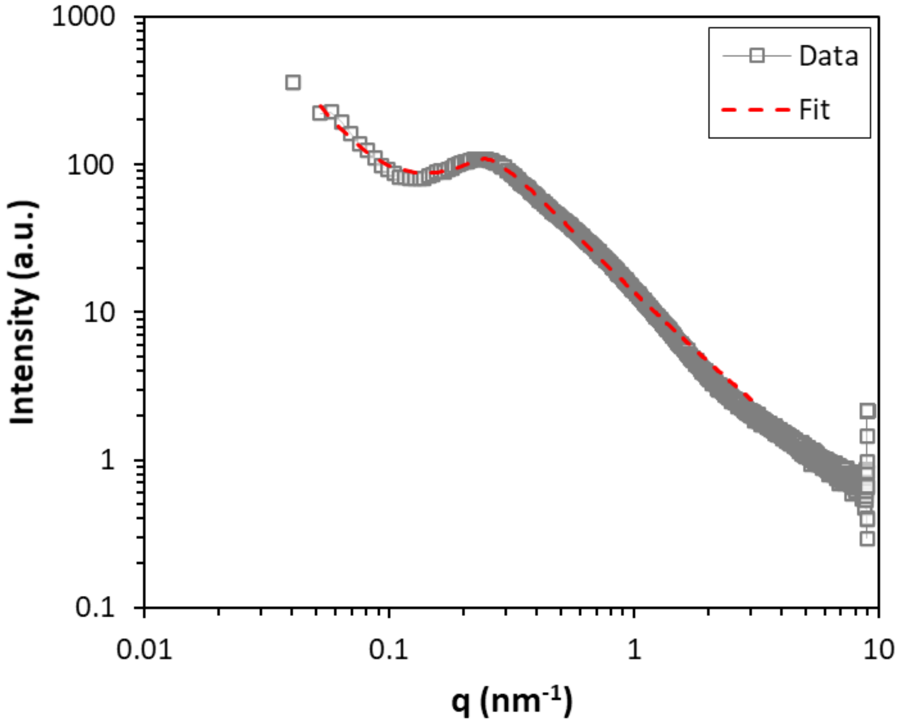

Figure S4. Demonstration of fitting dispersion SAXS data of 1 wt% 790 EW C4 in 50 wt% nPrOH (balance water) at 25°C with the model of Hammouda et al. (**eq S3**).

Table S3. Fitting parameters of the model of Hammouda et al. (**eq S3**) from the example fit shown in **Figure S4**.

A_{H2}	D	A_{H2}	Correlation Length, ξ (nm)	q_{max} (nm^{-1})	m	BG
0.11 ± 0.02	2.55 ± 0.05	105 ± 1	5.4 ± 0.1	0.248 ± 0.001	1.37 ± 0.03	0 ± 0

References

(1) Yeo, R. S. Solubility Parameter of Perfluorosulfonated Polymer. In *Perfluorinated Ionomer Membranes*; 1982; Vol. 180, pp 65–77.

(2) Hammouda, B.; Horkay, F.; Becker, M. L. Clustering and Solvation in Poly(Acrylic Acid) Polyelectrolyte Solutions. *Macromolecules* **2005**, *38* (5), 2019–2021. https://doi.org/10.1021/ma047960g.

(3) Teubner, M.; Strey, R. Origin of the Scattering Peak in Microemulsions. *The Journal of Chemical Physics* **1987**, *87* (5), 3195–3200. https://doi.org/10.1063/1.453006.
